# Assessing water resource system vulnerability to unprecedented hydrological drought using copulas to characterize drought duration and deficit

**DOI:** 10.1002/2015WR017324

**Published:** 2015-11-16

**Authors:** Edoardo Borgomeo, Georg Pflug, Jim W. Hall, Stefan Hochrainer‐Stigler

**Affiliations:** ^1^ Environmental Change Institute, University of OxfordOxfordUK; ^2^ Risk, Policy and Vulnerability ProgramInternational Institute for Applied Systems AnalysisLaxenburgAustria; ^3^ Department of Statistics and Operations ResearchUniversity of ViennaViennaAustria

**Keywords:** hydrological drought, copula, hydrological persistence, vulnerability based, drought vulnerability, climate change robustness

## Abstract

Global climate models suggest an increase in evapotranspiration, changing storm tracks, and moisture delivery in many parts of the world, which are likely to cause more prolonged and severe drought, yet the weakness of climate models in modeling persistence of hydroclimatic variables and the uncertainties associated with regional climate projections mean that impact assessments based on climate model output may underestimate the risk of multiyear droughts. In this paper, we propose a vulnerability‐based approach to test water resource system response to drought. We generate a large number of synthetic streamflow series with different drought durations and deficits and use them as input to a water resource system model. Marginal distributions of the streamflow for each month are generated by bootstrapping the historical data, while the joint probability distributions of consecutive months are constructed using a copula‐based method. Droughts with longer durations and larger deficits than the observed record are generated by perturbing the copula parameter and by adopting an importance sampling strategy for low flows. In this way, potential climate‐induced changes in monthly hydrological persistence are factored into the vulnerability analysis. The method is applied to the London water system (England) to investigate under which drought conditions severe water use restrictions would need to be imposed. Results indicate that the water system is vulnerable to drought conditions outside the range of historical events. The vulnerability assessment results were coupled with climate model information to compare alternative water management options with respect to their vulnerability to increasingly long and severe drought.

## Key Points

- Used copulas to flexibly model temporal dependence in streamflow sequences
- Assessed impact of hydrological persistence on water system vulnerability
- Tested the robustness of alternative options to drought duration and deficit

## 1 Introduction

Drought is one of the most serious hazards faced by water resource supply systems. Water managers have traditionally planned their systems so as to be able to maintain supply through a drought of a given severity, often the worst on record [*Watts et al*., 2012]. The projected intensification of the hydrological cycle and drought under climate change [*Huntington*, 2006; *Sheffield and Wood*, 2008; *Cayan et al*., 2010; *Prudhomme et al*., 2014] means that water resources managers need to improve their understanding of the vulnerability of their systems to changing and possibly intensifying drought conditions and to develop robust management options to deal with longer and more intense droughts [*Forzieri et al*., 2014].

Analysis of water resource system vulnerability to drought is challenging because historical observations are often too short to capture significant drought events. To overcome this challenge, vulnerability to drought has been studied using stochastic streamflow models [*Jinno*, 1995; *Cancelliere et al*., 1998, 2009] and paleo‐reconstructed data [*Prairie et al*., 2008; *Tingstad et al*., 2013; *Ghile et al*., 2014a]. Output from climate models has also been used to project future water availability [e.g., *Manning et al*., 2009] and hydrological drought occurrence in the future [*Burke and Brown*, 2010; *Rahiz and New*, 2013; *van Huijgevoort et al*., 2014].

Studies investigating vulnerabilities to climate change in the water sector have focused on changes in mean and seasonality, for instance, by applying change factors to hydroclimatological variables [e.g., *Ng et al*., 2010; *Borgomeo et al*., 2014; *Hall and Borgomeo*, 2013], and on changes in interannual variability [e.g., *Steinschneider et al*., 2015], without specifically examining the role of monthly hydrological persistence on water resource vulnerability. The streamflow sequences generated with these approaches may contain droughts which are more intense than the ones found in the historical record owing to the increase in temperature and, subsequently, evaporation projected by climate models. However, change factor approaches assume constant interannual variability in the future, an assumption that is questionable because it implies that persistent multiyear droughts would not be any more likely than they are in the historical record. Increasing evapotranspiration, changing storm tracks, and moisture delivery patterns may mean that unprecedented droughts may occur in the future.

Several studies have highlighted the limited skill of climate models in simulating variables relevant for water resources management and drought vulnerability assessment, such as low‐frequency rainfall variability [*Johnson and Sharma*, 2009; *Johnson et al*., 2011; *Rocheta et al*., 2014; *Tallaksen and Stahl*, 2014]. It seems, therefore, that assessments of the response of water resource systems to climate change based on downscaled scenarios from GCMs might underestimate the occurrence of persistent streamflow anomalies which may materialize in multiyear droughts *Hall et al.* [2012]. The diversity of drought conditions encountered in the paleoclimatic record [*Tingstad et al*., 2013; *Ault et al*., 2013 *; Cook et al*., 2014; *Patskoski and Sankarasubramanian*, 2015] and the risk of multiyear megadroughts [*Overpeck*, 2013] imply that, independent of climate change, future drought conditions may go well beyond the drought variability currently observed in the historical streamflow and precipitation records.

In a changing climate, changes in atmospheric circulation patterns and in the frequency of cyclic climate phenomena, such as El Niño Southern Oscillation (ENSO), could lead to longer or more intense droughts. Climate model experiments suggest an increased ENSO frequency and intensity under increasing greenhouse‐gas concentrations [*Timmermann et al*., 1999; *Cai et al*., 2014]. The well‐known influence of ENSO on regional hydrologic variability and drought in many parts of the world (e.g., Pacific U.S. Northwest [*Piechota and Dracup*, 1996], Philippines [*Jaranilla‐Sanchez et al*., 2011], eastern Australia [*Verdon et al*., 2004], and the Iberian Peninsula [*Vicente‐Serrano*, 2005]) means that changes in ENSO characteristics could have far‐reaching implications for drought occurrence and water supply security around the world. Occurrence and variability of other climate phenomena known to be associated with hydrological persistence and drought, such as atmospheric blocking and monsoon circulation, could also change as a result of increasing sea surface temperatures (SST) and set the stage for periods of extended or acute precipitation and streamflow anomalies [*Sylla et al*., 2010; *Trenberth and Fasullo*, 2012].

Similarly, other important persistent modes of climate variability, such as the Atlantic Multidecadal Oscillation and the Pacific Decadal Oscillation, have been related to drought conditions in some parts of the world [*Enfield et al*., 2001; *McCabe et al*., 2004; *Nigam et al*., 2011]; yet the uncertainty in predicting their present and future occurrence and variation [*Mantua and Hare*, 2002; *MacDonald and Case*, 2005; *Wen et al*., 2014] means that it is difficult for water managers to assess the impact of these climate fluctuations on water supply security.

The points discussed above—the weakness of climate models in representing persistence [*Rocheta et al*., 2014], the evidence from the paleoclimatic record suggesting the possibility of long multiyear droughts [*Overpeck*, 2013], the risk that climate change may bring about changes in atmospheric circulation, and the difficulty to predict occurrence of persistent modes of climate variability that lead to drought—mean that water managers need new techniques to test water resource systems' vulnerability to changing levels of hydrological persistence and to identify management options for dealing with multiyear drought conditions. In this paper, we present a vulnerability‐based approach to test the response of water resource systems to intense and persistent drought conditions. Vulnerability is here considered as “biophysical” vulnerability [*Brooks*, 2003] and is expressed as a metric of the damage experienced by the system in response to a hazard, in this case drought. In urban water supply systems, the damaging event of concern is severe water shortage resulting in regular or total cuts of water supply. A similar metric could be used for irrigation water supply systems.

Vulnerability‐based, “scenario‐neutral,” and “decision‐scaling” approaches have been recognized as an alternative to typical top‐down climate model scenario‐driven methodologies for climate change impact assessment and adaptation planning [*Wilby and Dessai*, 2010; *Brown and Wilby*, 2012; *Nazemi and Wheater*, 2014]. Rather than seeking accurate predictions of future climate to identify the optimal policy for that particular set of predictions, these approaches aim to identify how much a given system responds to changes in relevant hydroclimatic variables and under which conditions critical system thresholds are exceeded [*Brown et al*., 2012; *Steinschneider and Brown*, 2013; *Turner et al*., 2014; *Whateley et al*., 2014; *Ghile et al*., 2014b]. The performance of different management strategies under a wide range of hydroclimatic conditions can be tested in these frameworks and robust management strategies may be identified [*Herman et al*., 2015]. In the water sector, these approaches have been applied to assess the robustness of fluvial flood safety margins in UK catchments [*Prudhomme et al*., 2010], to develop robustness indicators for water management in the Upper Great Lakes [*Moody and Brown*, 2013], to explore water resource system's vulnerability to changes in streamflow characteristics in southern Alberta [*Nazemi et al*., 2013] and to identify climate and land use change combinations that lead to critical hydrologic thresholds being exceeded [*Singh et al*., 2014].

In this paper, we employ a vulnerability‐based approach to quantify the response of London's urban water supply system to a wide range of drought conditions. We develop a stochastic streamflow generation technique to synthesize a large number of streamflow sequences with different degrees of drought durations and deficits, which we use to test the water resource system's vulnerability to drought and compare different water management options on the basis of their ability to reduce vulnerability to drought conditions. As in any vulnerability‐based work, we do not seek to provide probabilities of the conditions that lead the system into an unsatisfactory state [*Ramírez and Selin*, 2014]. The aim of this paper is to enhance understanding of vulnerability to drought in water resource systems and to explore assumptions with respect to monthly hydrological persistence which have not hitherto been well explored in climate change vulnerability assessment studies.

This paper puts forward two new contributions: (i) the use of copulas to model nonlinear temporal streamflow dependence at the monthly time scale and (ii) the analysis of water resource system vulnerability to changing monthly hydrological persistence characteristics. The paper is organized as follows. The study's rationale and streamflow generation method are presented in section 2. In section 3, the case study area is introduced, and in section 4, the method is applied to the case study area and results are presented. Section 5 discusses the advantages and limitations of our approach, and conclusions are presented in section 6.

## 2 Method

### 2.1 Rationale

Meteorological droughts result from lack of precipitation for an extended period of time [*Tallaksen and van Lanen*, 2004]. Hydrological droughts ensue from meteorological droughts; however, their onset and development is heavily influenced by catchment characteristics, especially evapotranspiration [*Van Loon and Laaha*, 2015]. Hydrological droughts are typically defined as periods where streamflow falls below a predefined threshold [e.g., *van Huijgevoort et al*., 2012; *Watts et al*., 2012]. In this study, we define drought using a monthly Q75 threshold (i.e., the flow exceeded 75% of the time). A drought initiates when the streamflow falls below the Q75 threshold and it terminates when the streamflow becomes greater than the monthly Q75 threshold. Drought duration (i.e., length of time spent below the threshold) and drought deficit (i.e., cumulative deviation between streamflow and threshold level) can be derived using this threshold level approach. For each drought event, we calculate the duration *L* as the cumulative length of time in months spent below the monthly varying threshold *q* for each drought event *j*. The deviation *d* at time *t* for each drought event can be calculated as the difference between the streamflow *x*(*t*) and the threshold *q*(*t*) [*Hisdal et al*., 2004; *Van Loon et al*., 2014]:

(1) $$d(t)\;=\;\{\begin{array}{l} q\left(t\right)-x\left(t\right)\;\;\;\;if\;x\left(t\right)<q(t)\;\\ \;0\;\;\;\;\;\;\;\;\;\;\;if\;x\left(t\right)\geq q(t)\; \end{array}\}$$

The deficit *D* for event *j* is given by:

(2) $${D_{j}}_{\;}=\;\sum_{t=1}^{L_{j}}d(t)$$

Drought duration and deficit have major effects on the performance of a water resource system during a drought, because a short intense drought (high drought deficit) may imply rapid reservoir drawdown rates but also a quick recovery, whereas long droughts imply less rapid reservoir drawdown but sustained low reservoir levels conditions. By synthesizing streamflow time series with different drought characteristics, we can evaluate the system's vulnerability to drought, assess the system's relative sensitivity to drought duration and deficit, and test the robustness of different management options.

Drought duration can be related to the temporal dependence structure of the streamflow time series. Streamflow observations are autocorrelated in time, a property known as hydrological persistence, and the strength of this dependence is of interest when modeling drought because, at the most basic level, a stronger dependence means that dry periods are more likely to be followed by dry periods and wet periods by wet periods [*Pelletier and Turcotte*, 1997]. Perturbing the temporal dependence structure of a streamflow time series therefore allows us to change its monthly hydrological persistence characteristics and generate droughts longer than the ones present in the historical record. Furthermore, streamflow series often show a stronger dependence between dry months than between wet months, that is, they show nonlinear temporal dependence. We employ a copula‐based approach to model and perturb this nonlinear temporal dependence between consecutive months in the streamflow series.

Drought deficit is a measure of the severity of a drought and can be related to the occurrence of sustained periods of very low flows in the time series. A short intense drought with high drought deficit will be observed when extremely low flow conditions persist for a short duration. To generate such rare events, it is essential to adopt a sampling strategy that specifically selects the lowest values from the streamflow distribution. To achieve this, we employ an importance sampling strategy—a technique that increases the probability of sampling low flows.

Next, we introduce a stochastic framework that synthesizes streamflow time series with different levels of drought duration and deficit. The marginal distributions of the streamflow for each month are generated by bootstrapping the historical data. The temporal dependence structure of the time series—controlling drought duration—is represented and perturbed using copulas, while the very low flow occurrence—controlling drought deficit—is controlled by combining the copula with importance sampling.

### 2.2 Representing Temporal Dependence Using Copulas

#### 2.2.1 Archimedean Copulas

Several methods exist to represent the temporal dependence structure of hydrological time series. Examples include autoregressive moving average (ARMA) models [e.g., *O'Connell*, 1971; *Salas and Obeysekera*, 1982; *Stedinger et al*., 1985], fractionally differenced ARIMA models [*Montanari et al*., 1997], nonparametric methods [e.g., *Lall and Sharma*, 1996; *Sharma et al*., 1997], or nonparametric approaches combining paleoreconstructed data with K‐nearest neighbor bootstrap [e.g., *Prairie et al*., 2008]. Methods based on copulas have been proposed to model dependence of hydrological variables [*Salvadori and De Michele*, 2004]. Compared to other methods based on classical autocorrelation or spectral analysis and wavelet transforms [e.g., *Sen et al*., 2003], copula methods have the advantage of not requiring the streamflow data to be transformed to normality and of allowing for the modeling of nonlinear dependence. We therefore use copulas to model and perturb the temporal dependence of the monthly streamflow time series as described below.

Consider two continuous random vectors of streamflow monthly totals *Y_i−_*_1_ and *Y_i_* for two different consecutive months *i −* 1 and *i* observed over *n* years. The month vectors *Y_i−_*_1_ and *Y_i_* have marginal cumulative empirical distribution functions ${\hat{F}}_{Y_{i-1}}(y_{i-1})$ and ${\hat{F}}_{Y_{i}}(y_{i})$ and probability density functions $f_{Y_{i-1}}(y_{i-1})$ and $f_{Y_{i}}(y_{i})$, respectively.The joint cumulative distribution function *H*(*y_i−_*_1_, *y_i_*) of any pair of consecutive months (*Y_i−_*_1_, *Y_i_*) can be expressed as:

(3) $$H(y_{i-1},\;y_{i})\;=\;C_{\theta }\{{\hat{F}}_{Y_{i-1}}(y_{i-1}),\;{\hat{F}}_{Y_{i}}(y_{i})\}\;=\;C_{\theta }(u,\;v)$$

where *C*:[0,1]^2^→[0,1] is the copula function that captures the dependence between *Y_i−_*_1_ and *Y_i_, θ* is the copula parameter that measures the dependence between the marginal CDFs, and *u* and *v* are defined as realizations of the random variables *U* = ${\hat{F}}_{Y_{i-1}}(y_{i-1})$ and *V* = ${\hat{F}}_{Y_{i}}(y_{i})$. The copula parameter *θ* changes throughout the year for different pairs of consecutive months. Given Sklar's theorem, copulas are invariant under strictly increasing transformations of *X* and *Y*, which means that the pair (*U*, *V*) has the same copula *C* as (*Y_i−_*_1_, *Y_i_*) [*Salvadori and De Michele*, 2004].

Many different types of copula structures *C* exist. In this study, we considered two types of Archimedean copulas—Clayton and Frank—to model the consecutive month‐to‐month dependence of the streamflow time series. The Clayton copula is asymmetric, meaning that it exhibits greater dependence for values at the lower tail of the distribution. The Frank copula, on the other hand, is symmetric (i.e., it shows the same dependence at both ends of the distribution). Another commonly used Archimedean copula, the Gumbel copula, was not considered here because it exhibits greater dependence in the upper tail of the flow distribution, whereas our focus is more on correctly representing dependence at the lower tail of the flow distribution where low flows occur. We chose to employ Archimedean copulas because they allow for the modeling of the month‐month dependence with only one parameter and because they are well suited for modeling dependence of nonnormal variables, such as monthly streamflow volumes. Archimedean copulas are the most popular copula family used in hydrology [*Prakash Khedun et al*., 2014] and have been widely applied to model dependence between hydrological variables [e.g., *Wang et al*., 2009; *Ghosh*, 2010; *Maity et al*., 2013; *Nazemi et al*., 2013; *Prakash Khedun et al*., 2014; *Timonina et al*., 2015].

Archimedean copulas are defined as follows:

(4) $$C\left(u,v\right)\;=\;\varphi^{-1}\left[\varphi \left(u\right)\;+\;\varphi \left(v\right)\right]$$

where φ is a continuous strictly decreasing generator function from [0,1] onto [0, ∞].

Amongst the large number of copulas in the Archimedean family, we examine the Clayton and Frank copulas. The Clayton copula is defined as:

(5) $$C_{\theta }(u,v)\;=\;\max\;\left({\left[u^{-\theta }+\;v^{-\theta }-1\right]}^{-1/\theta },\;0\right),\;\theta \;\in \;\left[-1,\infty \right]$$

with generator $\varphi (t)=\;\frac{1}{\theta }\left(t^{-\theta }-1\right)$.

The Frank copula is defined as:

(6) $$C_{\theta }(u,v)\;=\;-\frac{1}{\theta }\ln\left(1+\;\frac{\left(\exp\left(-\theta u\right)-1\right)\left(\exp\left(-\theta v\right)-1\right)}{\exp\left(-\theta \right)-1}\right),\;\theta \;\in \;R$$

With generator $\varphi (t)=\;\ln\left(\frac{\exp\left(-\theta t\right)-1}{\exp\left(-\theta \right)-1}\right)$.

The copula parameter *θ* was estimated using the canonical maximum likelihood method [e.g., *Vandenberghe et al*., 2010].

#### 2.2.2 Goodness‐Of‐Fit and Copula Selection

Graphical and formal tests were used to evaluate the estimated copula structures and to select the most suitable copula for modeling the month‐to‐month dependence. To graphically assess the goodness of fit, we plotted the [0,1] normalized empirical cumulative distribution *K_n_* of the pseudo‐observations *w_i_* defined as [*Vandenberghe et al*., 2010]:

(7) $$K_{n}\left(b\right)=\;\frac{1}{n}\sum_{i=1}^{n}I\left(w_{i\;}\leq b\right),\;b\;\in [0,\;1]$$

where

(8) $$w_{i\;}=\;\frac{1}{n}\;\sum_{j=1}^{n}I\left({{(Y}_{i-1})}_{j}<\;{\left(Y_{j-1}\right)}_{i}\;\cap \;{\left(Y_{i}\right)}_{j}<\;{\left(Y_{j}\right)}_{i}\right)$$

where *i* is the indicator function. For each data point (*Y_i_*, *Y_i_*_−1_), one generates a new pseudo‐observation *w_i_* by counting how may data points (*Y_j_*, *Y_j_*_−1_) are smaller or equal in both components and dividing the result by *n*. The empirical distribution function of the *w_i_* is called *K_n_*. We plot this normalized empirical cumulative distribution *K_n_* against the theoretical distribution *K* which for the two Archimedean copulas with generator *φ* is defined as:

(9) $$K\left(t\right)\;=\;t\;-\;\frac{\varphi \left(t\right)}{\varphi^{'}\left(t\right)},\;for\;0\;<\;t\;\leq \;1$$

where

(10) $$\varphi '\left(t\right)=\frac{d}{d\left(t\right)}\varphi (t)$$

and where (*u*, *v*) are sampled from the theoretical copula *C_θ_* with the estimated parameter *θ*. If the curves of *K*(*t*) and *K_n_*(*t*) coincide, then there is a good fit between the data and the estimated copula.

The goodness of fit of the tested copulas was also assessed using two popular formal goodness‐of‐fit measures: the rank‐based version of the Cramér‐von Mises and the Kolmogorov Smirnov statistics [*Genest et al*., 2009; *Vandenberghe et al*., 2010; *Maity et al*., 2013]. These measures are based on the distance between the empirical copula *C_n_*—the empirical distribution of the rank transformed monthly data—and the theoretical copula *C_θ_*, constructed by evaluating the Frank and Clayton copulas with the estimated parameters. Low values of these statistics indicate a good fit of the copula models. For a detailed explanation of how to calculate these statistics, the reader is referred to *Genest et al*. [2009].

### 2.3 Streamflow Sampling

Monthly streamflow time series were generated by bootstrapping the observed monthly streamflow data. Bootstrapping is a common data resampling strategy, which is capable of simulating the probability distribution of any random variable without making any assumption about underlying distributions and without estimating parameters. As a time series model, the bootstrap simply amounts to resampling, with replacement, from the empirical distribution of the flow totals observed for each month. Bootstrap techniques have been widely applied to resample hydrologic time series [e.g., *Lall and Sharma*, 1996; *Vogel and Shallcross*, 1996]. In this study, the temporal structure of the time series is preserved by conditioning the bootstrap sampling via the copula structure. The flow at each month can be generated using a procedure of the type outlined by *Salvadori and De Michele* [2007].

Given the flow realization for the previous month [*Y_i−_*_1_ = *y_i−_*_1_] and its associated empirical distribution ${\hat{F}}_{Y_{i}}^{-1}$, the flow *y_i_* is generated as follows:

(11) $$y_{i}\;=\;{\hat{F}}_{Y_{i}}^{-1\;}\left[C_{y_{i-1}}^{-1}(v)\right]$$

where

(12) $$C_{y_{i-1}}\left(v\right)=C_{1}{(\hat{F}}_{Y_{i-1}}\left(y_{i-1}\right),\;v)$$

with

(13) $$C_{1}\left(u,\;v\right)=\frac{\partial }{\partial u}C(u,v)$$

We note that as ${\;\hat{F}}_{Y_{i}}^{\;}$ is an empirical distribution function, its inverse ${\;\hat{F}}_{Y_{i}}^{-1\;}$ will always yield a flow observation, so the approach is effectively bootstrapping the observed series. In this streamflow resampling scheme, an observation for month *i* is sampled from its empirical distribution function ${\;\hat{F}}_{Y_{i}}^{-1\;}$ according to the random variate *v*: [0,1]. *v* is generated with the copula parameter *θ_i_* for the month pair in question conditional on *u*:

(14) $$v={\left[1+\;u^{-\theta_{i}}\cdot \left(z^{\left(-\;\frac{\theta_{i}}{1+\;\theta_{i}}\right)}-1\right)\right]}^{-\frac{1}{\theta_{i}}}$$

where *u* is a realization of the random variable *U* = ${\hat{F}}_{Y_{i-1}}(y_{i-1})$ from the previous month and *z* is a uniform random variable on the interval (0,1).

Bootstrapping is a convenient way of generating the marginal distributions for each month; however, it is limited when modeling rare events like droughts because it may not give enough samples from the low flow region of the monthly flow distribution, which are the events of most interest. This means that using the bootstrap method and perturbing the copula dependence parameter may not have the potential to generate time series with very large drought deficit and thus may not allow for a full characterization of the drought duration‐deficit space.

To overcome this limitation and generate short and intense drought events with high deficits, we employed importance sampling. In the importance sampling, samples are weighted by:

(15) $$s\left(r\right)=\sqrt{\frac{n}{r}}$$

where *r* is the flow rank and *n* the number of observations. The weight *s* is applied to the empirical distribution $f_{Y_{i}}(y_{i}$) to increase the probability density in the lower tail, thus increasing the probability of consecutively sampling low flows.

The importance sampling is applied to month *i* only when the flow realization in the previous month *i−*1 is less than or equal to a predefined threshold *T* (Q90 in our example). When this condition is satisfied, the flow for month *i* is generated following the same procedure as equations (1), (2), (3), (4), (5), (6), (7), (8), (9), (10), (11), (12), (13) above; however, the flow distribution $f_{Y_{i}}(y_{i}$) is weighted by *s*.

The importance sampling ensures that low flows (i.e., flows with small ranks) have a higher chance of being selected than high flows. This sampling strategy essentially leads to two or more of the lowest flows observed in the historical record to occur consecutively, generating a drought with a higher deficit than the droughts in the observed record.

### 2.4 Streamflow Scenario Generation

**Figure 1:**
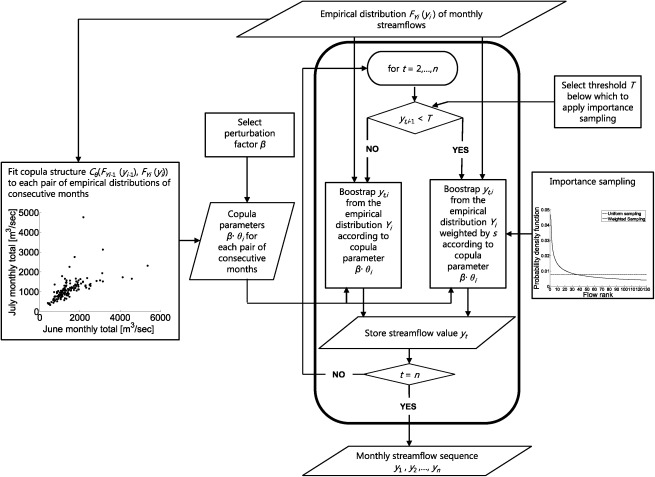
Flowchart of the synthetic streamflow generation approach combining copulas with importance sampling.

In this section, we provide a step by step summary of the streamflow generation algorithm to demonstrate how the copula is coupled with the importance sampling strategy. A flowchart of the synthetic streamflow generation method is shown in Figure 1.

1. Fit a copula structure (e.g., Frank, Clayton, etc.) $C_{\theta }\left(F_{Y_{i-1}}\left(y_{i-1}\right),\;F_{Y_{i}}\left(y_{i}\right)\right)$ with parameter *θ_i_* to each pair of marginal distributions $\left(F_{Y_{i-1}}\left(y_{i-1}\right),\;F_{Y_{i}}\left(y_{i}\right)\right)$ of total monthly flows for consecutive months *i* − 1 and *i* in the historical streamflow time series;
2. Generate a monthly streamflow sequence *y*_1_, *y*_2_,…, *y_n_*. For each time step, *t =* 2*,…,n*:
  - Select the fitted copula $C_{\theta }\left(F_{Y_{i-1}}\left(y_{i-1}\right),\;F_{Y_{i}}\left(y_{i}\right)\right)$ and parameter *θ_i_* for the consecutive month pair in question;
  - Generate a random variate *v*: [0,1] according to a copula with parameter *βθ_i_* conditioned on the streamflow from the previous month *y_t−_*_1,__*i*_ (equation (14)), where *β* is a perturbation factor which alters the temporal dependence structure of the sequence;
  - Select a threshold value *T* below which importance sampling is applied;
  - If *y_t−_*_1,__*i*_ < *T*,
    - Bootstrap a random variate *y_t,i_* with replacement from the empirical distribution of the month *Y_i_* weighted by *s* according to the random variate *v*.
  - If *y_t−_*_1,__*i*_> *T*,
    - Bootstrap a random variate *y_t,i_* with replacement from the empirical distribution of the month *Y_i_* according to the random variate *v*.
3. The resulting monthly streamflow sequence is *y*_1_, *y*_2_,…, *y_n_*.

## 3 Case Study

### 3.1 London Urban Water Supply System

We apply the method presented above to assess the response of the London urban water supply system to different drought duration and deficit conditions. The London urban water supply system is located in the Thames River Basin, south‐east of England. The Thames River Basin is a heavily urbanized basin with a population of around 12 million, dominated by the city of London. The Thames River Basin is one of the driest river basins in the UK, receiving an annual average rainfall of 690 mm [*Environment Agency*, 2014], and has been classified as being under severe water stress because demand is a high proportion of current effective rainfall [*Environment Agency*, 2013]. Studies examining the impacts of climate change in the area suggest potential reductions in the basin's water availability in the future [*Manning et al*., 2009; *Diaz‐Nieto and Wilby*, 2005].

Water supply in the Thames River Basin is managed by private water utilities at a water resource zone level, defined as a zone where water users experience the same risk of supply failure. Our case study is based on a water resource system model of the London water resource zone, the largest water resource zone in the Thames River Basin, which supplies water to approximately 7 million people.

**Figure 2:**
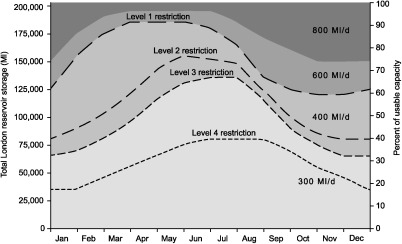
The Lower Thames Operating Agreement showing storage levels that trigger demand restrictions and environmental flow requirements.

The London water resource zone is a conjunctive use system supplied by surface water abstractions from the River Thames and groundwater abstractions from the Chalk Aquifer, which, respectively, account for 80% and 20% of total supply. Abstractions from the River Thames to support London's household demand take place upstream of Kingston. Abstracted surface waters are stored in a system of pumped storage reservoirs, which are used to supply London's urban water demands. Depending on the percentage of raw water storage in London's reservoirs, demand restrictions are imposed on water users according to the Restriction Levels shown in Figure 2, which result in the expected demand reductions shown in Table 1.

**Table 1:** Demand Restriction Levels Corresponding to the Reservoir Levels in Figure 2 and Corresponding Expected Demand Reductions [*Thames Water*, 2014]

Level of Restriction	Water Use Restrictions	Expected Demand Reduction (Cumulative)
Level 1	Intensive media campaign	2.2%
Level 2	Sprinkler/unattended hosepipe ban, enhanced media campaign	9.1%
Level 3	Temporary use ban	13.3%
Level 4	Emergency Drought Order for standpipes and rota cuts	31.3%

Minimum flows for environmental conservation are protected through the Lower Thames Operating Agreement shown in Figure 2, which sets the environmental flow requirements based on the percent of raw water storage in London's reservoirs. As reservoir levels drop, environmental flow requirements are gradually reduced to a minimum of 300 ML/d, thus allowing for continued abstraction during periods of drought. The London urban water supply system is also served by three strategic options, a 150 ML/d desalination plant and an artificially recharged aquifer, with a maximum output of 130 ML/d [*Thames Water*, 2014], which are activated to augment water supply during drought. These supply‐side options are activated when the naturalized flow at Kingston goes below 3000 ML/d on average for 10 consecutive days and when the reservoir levels fall below the 800–600 ML/d line in Figure 2. A third option consists of a groundwater scheme which can provide an extra water supply of 66 ML/d which is discharged directly into the River Thames upstream of Kingston [*Thames Water*, 2014]. This option is activated when the raw water storage in the reservoir falls below the Level 2 trigger in Figure 2.

The Thames River Basin has been affected by four major droughts in the last 100 years: 1920/1921; 1933/1934; 1943/1944; 1975/1976 [*Thames Water*, 2013]. These droughts resulted from prolonged periods—from 12 to 18 months—of below average precipitation and consecutive dry winters, which significantly reduced water availability leading to the introduction of drought mitigation measures including water use restrictions [*Marsh et al*., 2007; *Thames Water*, 2013].

### 3.2 Water Resource System Model

The London urban water supply system was modeled using the eWater Source IMS platform [*Carr and Podger*, 2012; *Welsh et al*., 2013]. eWater Source employs a node‐link structure to conceptualize water resource systems. Nodes can be used to represent streamflow gauges, confluences, groundwater abstraction points, reservoirs, and water users. All nodes are interconnected by links.

The model was set up to represent urban water and environmental demands as distinct water users and London's reservoirs as a single large reservoir representing their total combined capacity. To calculate how water is released from the reservoir to meet the environmental and urban water demands and simulate the operation of the water supply system at each time step, we employ rule‐based ordering, where water is allocated following a set of specified instructions. Environmental demands are a function of the percentage of the raw water storage in the reservoir, as displayed in Figure 2.

Household water demand was modeled using population and per capita consumption data for 2014 used by the local water utility company for planning purposes [*Thames Water*, 2014] and was considered to be constant through the year and across all simulations. This assumption is justified by studies of domestic water use pattern in London, which indicate that water consumption is not very sensitive to temperature [*Herrington*, 1996; *HR Wallingford*, 2012]. The system specifications, including the reservoir restriction levels shown in Figure 2 and the strategic schemes, were incorporated in the water resource system model. A proactive demand management strategy was implemented in the model, whereby demand was reduced by the values shown in Table 1 every time simulated reservoir levels fell below one of the four restriction thresholds in Figure 2.

The simulated monthly streamflow series were fed into the water resource system model at the Kingston inflow node. Groundwater supply was set to a constant value equal to the dry year deployable output, which is defined by the local water utility company as the maximum rate at which groundwater sources can supply water through a dry period [*Thames Water*, 2014].

### 3.3 Defining Water Resource System Thresholds

Several different water resource system vulnerability indicators and measures exist such as the ones proposed by *Hashimoto et al*. [1982], who defined vulnerability as a measure of the severity of the failure of the water supply system. Other examples of vulnerability indicators in the water sector include *Moody and Brown* [2012], who presented a series of stakeholder‐defined system thresholds for the Upper Great Lakes (USA) to measure water resource system performance over a wide range of climate conditions. In this study, we employ a stakeholder‐defined indicator based on reservoir levels. The use of a stakeholder‐defined indicator allows us to evaluate the vulnerability to drought in a way that is meaningful to water resources managers and decision‐makers.

The water utility company supplying London has stated that reaching the Level 4 restriction curve in Figure 2 implies severe restrictions on water use such as rota cuts and stand pipes [*Thames Water*, 2014]. These restrictions would have catastrophic impacts on London's economy, with some estimates suggesting 236–330 million British pounds per day [*Lambert*, 2015], and are therefore highly undesirable. In this study, we use this reservoir threshold to define the point at which the performance of the water resource system becomes unsatisfactory. The synthetic streamflow series are used as inflows to the water resource system model, which is run with a monthly time step. If in any simulation the reservoir levels fall below the Level 4 curve, the system is deemed to have reached an unsatisfactory state and the simulation is stopped.

## 4 Results

### 4.1 Validation of the Streamflow Generation Method

Monthly streamflow totals for the River Thames at Kingston, from 1883 to 2012, were used for the application of the proposed streamflow generation method. The degree of dependence between consecutive months in the historical series was quantified by computing the Spearman's rho and Kendall's tau coefficients, shown in Table 2. Higher values of these two coefficients indicating greater dependence were found for the summer months, further justifying the need for a mathematical approach like copulas that accounts for nonlinear dependence. This nonlinear temporal dependence is also illustrated in the scatterplots of streamflow observations for consecutive months (see supporting information). The greater dependence in the summer months can be ascribed to the greater base flow contribution to streamflow during the summer months in the Thames River Basin [*Bloomfield et al*., 2009].

**Table 2:** Parametric Measures of Dependence for Consecutive Months in the Monthly Total Streamflow Series (m^3^/s) Observed for the River Thames at Kingston 1883–2012

	January–February	February–March	March–April	April–May	May–June	June–July	July–August	August–September	September–October	October–November	November–December	December–January
Spearman's rho	0.58	0.61	0.65	0.74	0.81	0.8	0.85	0.79	0.74	0.69	0.66	0.64
Kendall's tau	0.41	0.44	0.48	0.56	0.63	0.62	0.66	0.6	0.56	0.5	0.48	0.44

The empirical copulas *C*(*Y_i−_*_1_,*Y_i_*) obtained for each month pair were compared with the fitted theoretical Clayton and Frank parametric copulas *C_θ_*(*Y_i−_*_1_,*Y_i_*) with the estimated parameters shown in Table 3 (see supporting information). A good fit is obtained for both Clayton and Frank copulas, and visual judgment alone cannot be used to select the appropriate copula.

**Table 3:** Estimated Clayton and Frank Copula Parameters for Consecutive Months in the Monthly Total Streamflow Series (m^3^/s) Observed for the River Thames at Kingston 1883–2012

	Month Pair											
	January–February	February–March	March–April	April–May	May–June	June–July	July–August	August–September	September–October	October–November	November–December	December–January
Clayton	1.75	1.55	1.97	3.04	3.28	3.08	3.95	3.08	2.29	2.26	2.06	2.09
Frank	4.30	4.65	5.28	6.71	8.22	8.23	9.40	7.77	6.50	5.60	5.25	5.61

The goodness‐of‐fit of the two copula structures was also assessed using two formal tests: the Cramér‐von Mises and the Kolmogorov‐Smirnov tests [*Genest and Favre*, 2007]. The *p* values obtained with the parametric bootstrap procedure introduced by *Genest et al*. [2009] for each copula structure are shown in Table 4. For both tests, *p* values for the Clayton copula are larger than the Frank copula, indicating that the Clayton copulas provide an adequate representation of the temporal dependence between consecutive month pairs [cf. *Durante and Salvadori*, 2010]. Given that in this study we are particularly interested in low flow conditions, the selection of the Clayton copula is also justified because this copula structure is appropriate for outcomes correlated at low values [*Dupuis*, 2007].

**Table 4:** Estimated *p* Values Based on 250 Bootstrap Sets of Goodness‐Of‐Fit Statistics for Clayton and Frank Copulas

Month Pair	Kolmogorov‐Smirnov		Cramér‐von Mises	
	Clayton	Frank	Clayton	Frank
January–February	0.44	0.21	0.26	0.12
February–March	0.5	0.34	0.35	0.32
March–April	0.46	0.2	0.42	0.12
April–May	0.41	0.21	0.38	0.11
May–June	0.4	0.21	0.4	0.32
June–July	0.4	0.32	0.37	0.3
July–August	0.48	0.28	0.45	0.33
August–September	0.55	0.28	0.52	0.28
September–October	0.43	0.3	0.38	0.28
October–November	0.48	0.32	0.38	0.32
November–December	0.51	0.23	0.5	0.29
December–January	0.51	0.21	0.42	0.27

**Figure 3:**
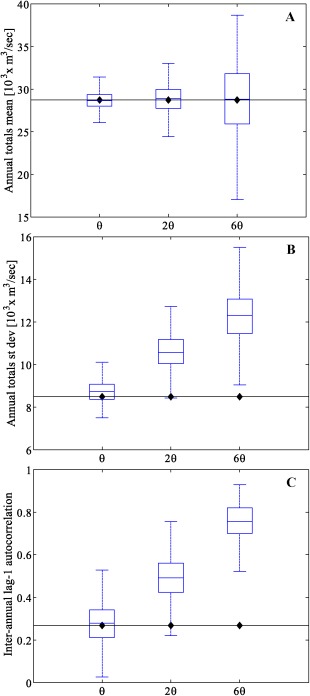
Box plots of the (a) mean, (b) standard deviation, and (c) interannual lag‐1 autocorrelation of 100 realizations of annual streamflows for the River Thames at Kingston simulated with three different values of the copula parameters *θ*. Horizontal lines represent the same statistics for observed annual streamflows.

To validate the streamflow generation algorithm and to test its effectiveness at preserving important properties of the observed streamflow time series, we generated 100 streamflow series each 100 year long using the historical *θ* parameters for the Clayton copula (Table 3) and the perturbed *θ* dependence parameters and computed basic annual and monthly statistics. At the annual time scale, the mean, standard deviation, and interannual lag‐1 autocorrelation characteristics of the observed time series are well preserved, as shown in Figure 3 for streamflow series generated with the historical *θ* parameters. When the *θ* parameters are perturbed (by factors *β* = 2 and *β* = 6 in this example), other streamflow series properties change as expected. The spread around the mean of the annual totals increases (Figure 3a). Increasing the dependence parameters *θ* also causes an increase in the interannual variability, as shown in the plot of the standard deviation of the annual totals (Figure 3b), and in the annual hydrological persistence, as shown in the plot of the lag‐1 autocorrelation (Figure 3c).

**Figure 4:**
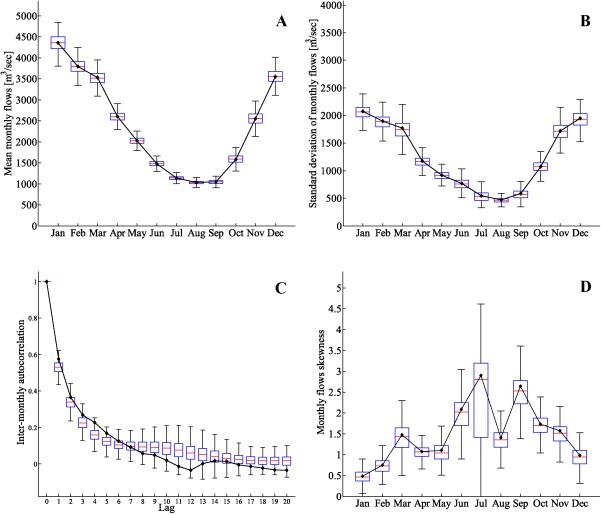
Boxplots of the (a) mean, (b) standard deviation, (c) autocorrelation function, and (d) skewness of 100 realizations of monthly streamflows generated with copula parameters *θ* for the River Thames at Kingston. Continuous lines with black dots represent the same statistics for the observed monthly flows.

At the monthly time scale, the mean, standard deviation, the autocorrelation structure, and the skewness of the data are well preserved for the streamflow series generated with the historical *θ* values (Figure 4). The autocorrelation function (Figure 4c) of the simulated sequences is comparable with the autocorrelation of the observed sequences, although it shows a slight underestimation for lags lower than seven which may be due to the fact that our copula model is only fitted to the first lag (i.e., only consecutive month pairs were used to fit the copula). The mismatch for lags greater than eight is not concerning given that the monthly autocorrelation for the Thames streamflow data at Kingston is only significant up to lag‐8 [*Borgomeo et al*., 2015].

**Figure 5:**
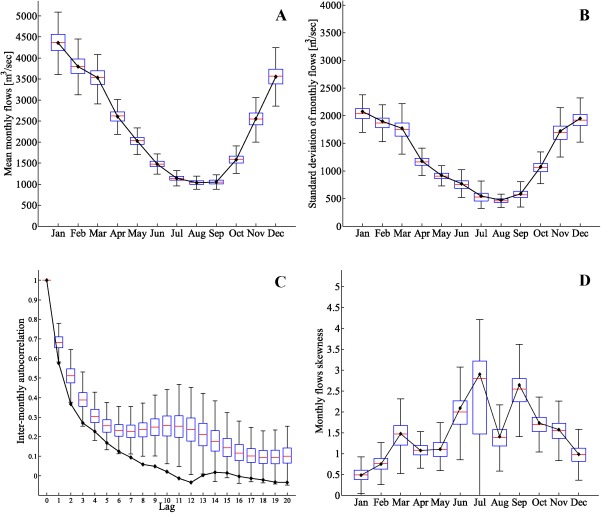
Boxplots of the (a) mean, (b) standard deviation, (c) autocorrelation function, and (d) skewness of 100 realizations of monthly streamflows generated with perturbed copula parameters 2*θ* for the River Thames at Kingston. Continuous lines with black dots represent the same statistics for the observed monthly flows.

Monthly statistics for streamflow time series generated by perturbing the *θ* parameter by a factor *β* = 2, equivalent to a doubling of the strength of the dependence between consecutive months, are shown in Figure 5. The monthly means (Figure 5a) and the intermonthly variability, displayed in the plot of monthly standard deviations (Figure 5b), are not changed significantly by the increase in *θ*. The intermonthly autocorrelation structure (Figure 5c) increases significantly, reflecting the increased temporal dependence imposed on the time series by the increase in the copula parameters *θ*. The skewness of the monthly data is generally well preserved (Figure 5d) and not altered by the *θ* parameters perturbation (Figure 5d).

**Figure 6:**
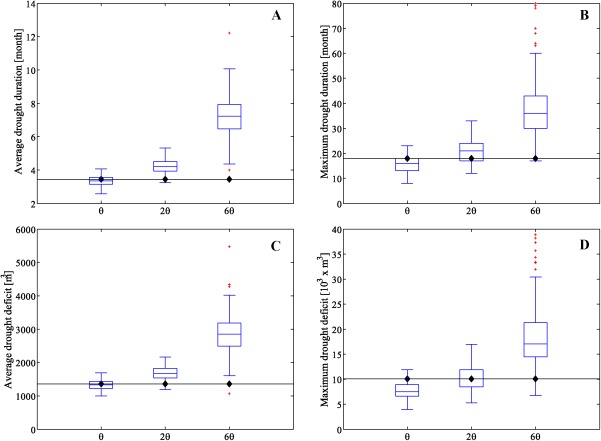
Boxplots of the (a) average drought duration, (b) maximum drought duration, (c) average drought deficit, and (d) maximum drought deficit of 100 realizations of monthly streamflows generated with three different values of copula parameters *θ* for the River Thames at Kingston. Continuous lines with black dots represent the same statistics for the observed monthly flows.

To establish a baseline [cf. *Li et al*., 2014] and test our streamflow generation method further, we plot the drought statistics for the observed monthly streamflows and the streamflows obtained with the historical and perturbed copula parameters *θ*. The average drought duration, maximum drought duration, average drought deficit, and maximum drought deficit (defined using a monthly Q75 threshold) for 100 realizations for each value of the copula parameters are shown in Figure 6. The drought statistics of the unperturbed simulated streamflows are comparable to the historical drought statistics, although the simulated sequences show a slight underestimation of the maximum drought duration and deficit. As expected, perturbing the dependence parameter increases the average and maximum drought duration (Figures 6a and 6b). The average and maximum drought deficit also increase as a result of the perturbation (Figures 6c and 6d).

These results demonstrate that our approach has the ability to correctly model the historical and perturbed dependence structure of monthly streamflow data and is capable of generating droughts with longer deficits and durations than the ones in the observed data. The changes in interannual variability and autocorrelation function are expected given that the copula parameter governs the temporal dependence structure of the time series, measured with the autocorrelation function, and the clustering of dry and wet periods, measured with the standard deviation of the annual totals.

### 4.2 Drought Scenarios

The streamflow generation method was used to generate 100 yearlong monthly streamflow time series of the River Thames at Kingston. The copula parameters in Table 3 were multiplied by a perturbation factor *β* with values ranging from 1 to 10 at 0.25 intervals to generate streamflow sequences with increasing levels of month‐to‐month dependence. For each perturbation, we generated 100,000 sequences employing the streamflow generation algorithm detailed in section 2.4 and Figure 1, using a monthly Q90 threshold for the importance sampling. On a 3.4 GHz processor, this streamflow scenario generation exercise took approximately 18 h of computing.

**Figure 7:**
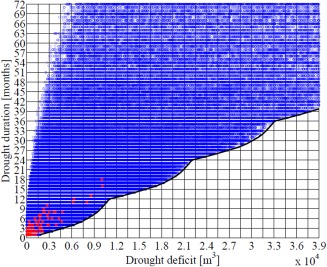
Scatterplot of drought duration and drought deficit statistics for the observed (red) and simulated (blue) monthly streamflow data.

We calculated the maximum drought duration in months and the maximum cumulative drought deficit in m^3^ for each synthetic sequence and then plotted the results in Figure 7. The drought duration and deficit for the drought events in the observed record are also shown in Figure 7 (red dots). A clear lower bound can be noticed in the drought duration‐deficit plot (black solid line in Figure 7). This lower bound is due to the definition of drought deficit (i.e., difference between the simulated flow and the monthly Q75) and the impossibility of simulating deficits larger than Q75 minus the minimum bootstrapped flow for that particular month. This is a common limitation of any bootstrapping method which only reproduces historical sample values [e.g., *Lall and Sharma*, 1996]; however, even if the streamflows had been sampled from a distribution, there would still be a lower bound in the drought duration‐deficit plot due to the impossibility of generating deficits larger than the seasonal Q75 threshold for any given month. An upper bound can also be noticed in the upper left corner of the drought duration‐deficit plot because of the difficulty of generating streamflow series that spend significant amount of time below the Q75 threshold (i.e., long duration) without accumulating any significant deficit (i.e., it is impossible to have a drought that lasts for a long time and does not accumulate any deficit).

To conduct the vulnerability analysis, we partitioned the drought duration‐deficit space into discrete intervals. We considered ranges between 0 and 4 × 10^4^ m^3^ with a 0.15 × 10^4^ m^3^ step for drought deficit and between 0 and 72 with a 3 months step for drought duration, for a total of 460 grid combinations. These ranges extend to about 4 times the maximum drought durations and deficits of the observed record, allowing us to test the system's response to conditions which significantly depart from the historical. The grid used to divide the drought duration‐deficit space is shown in Figure 7. For each grid cell, we randomly selected 100 simulations with the drought deficit and duration of that grid cell, for a total of 460,000 scenarios.

### 4.3 Vulnerability Assessment

The streamflow series with the selected drought duration‐deficit properties were used as inflow inputs to the London water supply system model. In the water resource system simulation, the initial time step coincided with the start of the drought event. All simulations started with the full reservoirs, which is typically the case in London at the end of the winter. For each grid cell, we counted the fraction of simulations (out of the total 100 simulations for each grid cell) that reached an unsatisfactory state. An unsatisfactory state is reached every time the reservoir levels in the simulation go below the Level 4 control curve (Figure 2). The fraction of simulations reaching an unsatisfactory state is used here as a measure of the system's vulnerability to different drought durations and deficits. This metric essentially uses a domain criterion to measure vulnerability [*Herman et al*., 2014, 2015], that is, it seeks to quantify the area of the drought deficit duration space where the system's performance meets the decision‐makers' requirements.

**Figure 8:**
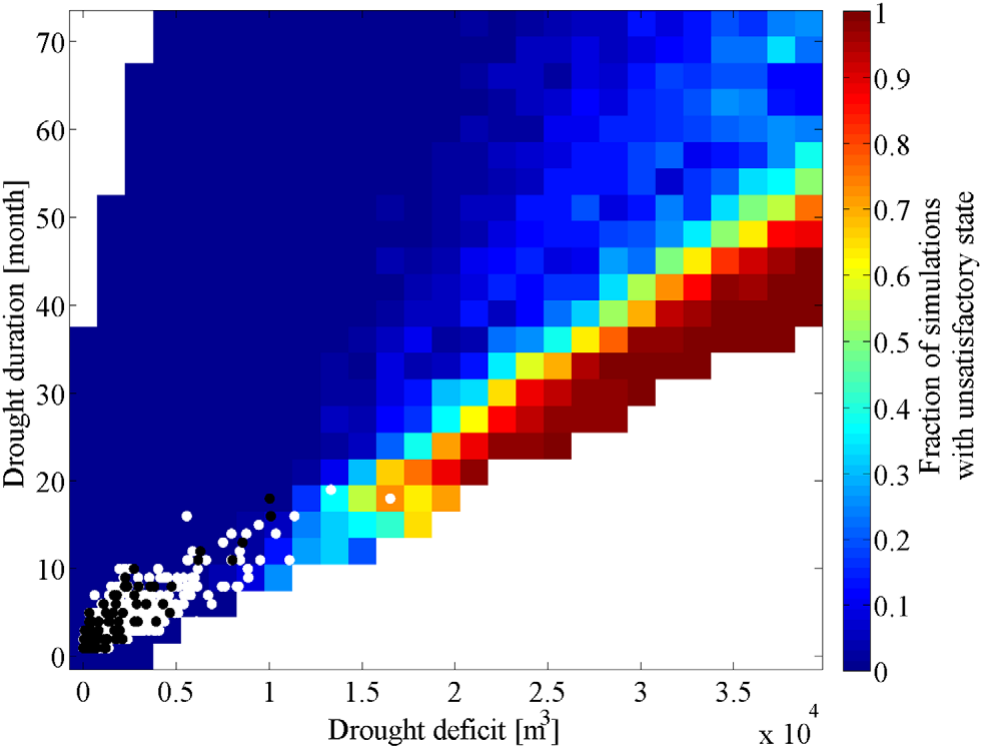
Fraction of water resource system simulations reaching an unsatisfactory state. Black dots represent historical drought conditions, white dots represent drought conditions projected by the 11 members of the Future Flows hydrology ensemble.

The water supply system's response to drought conditions can be visualized in Figure 8, which provides a vulnerability map of London's water resource system under different drought conditions. Figure 8 shows the fraction of runs reaching an unsatisfactory state (i.e., a Level 4 restriction) under a particular combination of drought duration and deficit given the current system's supply infrastructure and demands.

In general, the water resource system is more sensitive to drought deficit than drought duration, suggesting that the London water supply system is vulnerable to drought events with large deficits accumulating over a time period of 1 year or longer. The plot shows that a 2 yearlong drought with a cumulative deficit between 1.5 × 10^4^ and 2 × 10^4^ m^3^ could cause the system to reach an unsatisfactory state. The lower fraction of simulations reaching an unsatisfactory state in the top right corner of Figure 8 implies that the water resource system is less vulnerable to droughts where deficit slowly accumulates over long periods of time. This effect is due to the water resource system threshold we used to define vulnerability (section 3.3) and the threshold we used to define drought (section 2.1). Very large differences between the simulated streamflow and the Q75 threshold accumulating over a short period of time imply rapid reservoir drawdown, which results in the Level 4 curve being exceeded. A deficit accumulating over a longer period of time implies a streamflow sequence which is just below the Q75 threshold and which allows the reservoir levels to recover during the winter months, thus avoiding the Level 4 restriction curve. Future work should examine the vulnerability of water resource systems to metrics based on cumulative reservoir deficit rather than a fixed threshold.

The drought duration deficit response surface in Figure 8 can also be used as a drought management tool by water managers in the London water supply area. Ongoing droughts can be mapped onto this space and the implications of their development in terms of critical system thresholds assessed. For instance, if drought characteristics obtained from seasonal streamflow forecasts show that the system is heading toward one of the vulnerable regions, then water managers may decide to implement drought management measures and water use restrictions (e.g., a temporary use ban) to avoid reaching a Level 4 restriction [e.g., *Golembesky et al*., 2009].

The plausibility of the drought duration and deficit conditions in Figure 8 was assessed by plotting the drought durations and deficits of the historical streamflow series and of the streamflow projections for the Thames at Kingston from the Future Flows project [*Prudhomme et al*., 2013]. Future Flows consist of an 11 member ensemble of transient projections of streamflow time series up to 2098, each derived from simulated series from the Hadley Centre's regional climate model HadRM3‐PPE propagated through hydrological models [*Prudhomme et al*., 2013]. The drought durations and deficits calculated for these projections are shown as white dots in Figure 8, while the duration and deficit calculated for the historical streamflow data are shown as black dots in Figure 8. The Future Flows ensemble contains droughts with greater deficits than the historical, an effect that can be ascribed to the projected temperature and evaporation increases in the UK [*Murphy et al*., 2009; *Watts et al*., 2015] . However, Figure 8 shows that the Future Flows ensemble does not contain droughts which are longer than historical droughts, highlighting the limitations of hydroclimatic projection‐based assessments of drought vulnerability.

Figure 8 demonstrates a few important points. First, the comparison of the model runs with the historical data and the GCM‐based drought projections demonstrates that our approach can produce a much wider range of drought scenarios than that produced with climate models. Second, drought events with the same length but larger deficits than historical drought events could push the system into an unsatisfactory state. These conditions are plausible given that the most severe drought in the historical record lasted for about 18 months and had a cumulative deficit of about 1 × 10^4^ m^3^. Third, under one of the climate scenarios, the system has a high likelihood of reaching an unsatisfactory state, suggesting that the water resource system is vulnerable to changing drought characteristics as represented in the Future Flows projections. Based on this information, a risk‐averse water supply manager may deem unacceptable the conditions depicted in light‐blue, where about 30% of the simulations reach an unsatisfactory state, and decide that management actions (e.g., water transfers, enhanced leakage reduction) are needed to avoid severe water use restrictions.

### 4.4 Characterizing the Robustness of Alternative Water Management Options

The vulnerability metric based on the Level 4 restriction curve was used to characterize the robustness of three drought management options for London. These options were chosen for illustrative purposes only to show how our method enables water managers to compare options based on their robustness to unprecedented drought conditions. The importance of understanding the robustness of water management options to drought is considered as a critical test to guide the selection and judge the success of water management plans in the London water resource zone [*Thames Water*, 2014].

Three drought management options were considered: (i) a 65 ML/d interbasin transfer activated when reservoir levels drop below the Level 1 threshold in Figure 2; (ii) a 90 ML/d interbasin transfer activated when reservoir levels drop below the Level 1 threshold in Figure 2; (iii) enhanced leakage reduction and demand management achieving 9.1% and 13.3% expected demand reductions at Levels 1 and Levels 2, respectively (see Table 1). This latter drought management option corresponds to a scenario where water managers apply more stringent water use restrictions earlier during a drought than is currently done.

The water resource system model was modified to incorporate each one of the three management options and the simulations were run again. To compare the robustness of each alternative option, we identify their worst performance in increasing unlikely droughts, where the likelihood is estimated from the drought events contained in the Future Flows projections. The marginal distributions of the drought duration and deficit of the Future Flows were found to be exponentially distributed. The Gumbel copula was found to best represent the joint distribution of duration and deficit (see supporting information).

**Figure 9:**
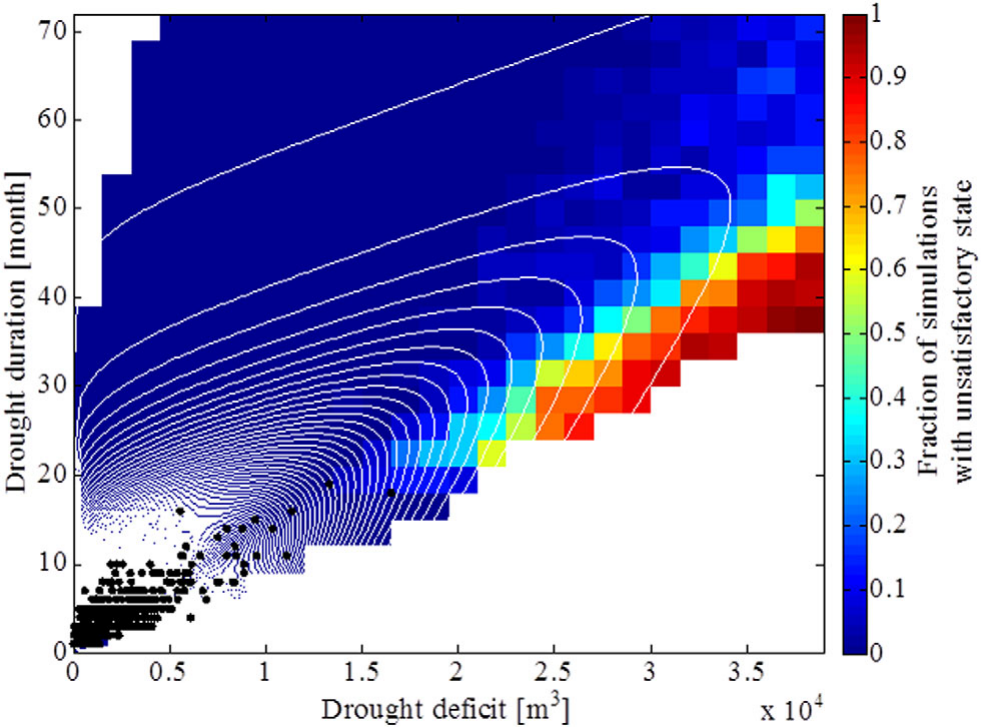
Joint density of duration and deficit obtained with the Gumbel copula (white contours) plotted over the fraction of simulations with unsatisfactory state for the enhanced demand reduction option. Black dots represent the drought events contained in the Future Flows projections.

The joint probability distribution obtained via the Gumbel copula is shown in Figure 9 and is given by [*Balakrishnan and Lai*, 2009]:

(16) $$p\left(L,\;D\right)\;=\;\exp\left[-{\left(e^{-\theta D}+e^{-\theta L}\right)}^{\frac{1}{\theta }}\right]$$

Where *L* is drought duration, *D* is drought deficit, and *θ* is the Gumbel copula parameter. We explore the sensitivity to increasingly severe combinations of drought duration and deficit by plotting the worst system performance *F_p′_* for different *p*′ values of the joint probability distribution:

(17) $$F_{p^{\prime }}=\underset{p(L,D)\geq p^{\prime }}{\max}\left\{F_{L,D}\right\}$$

**Figure 10:**
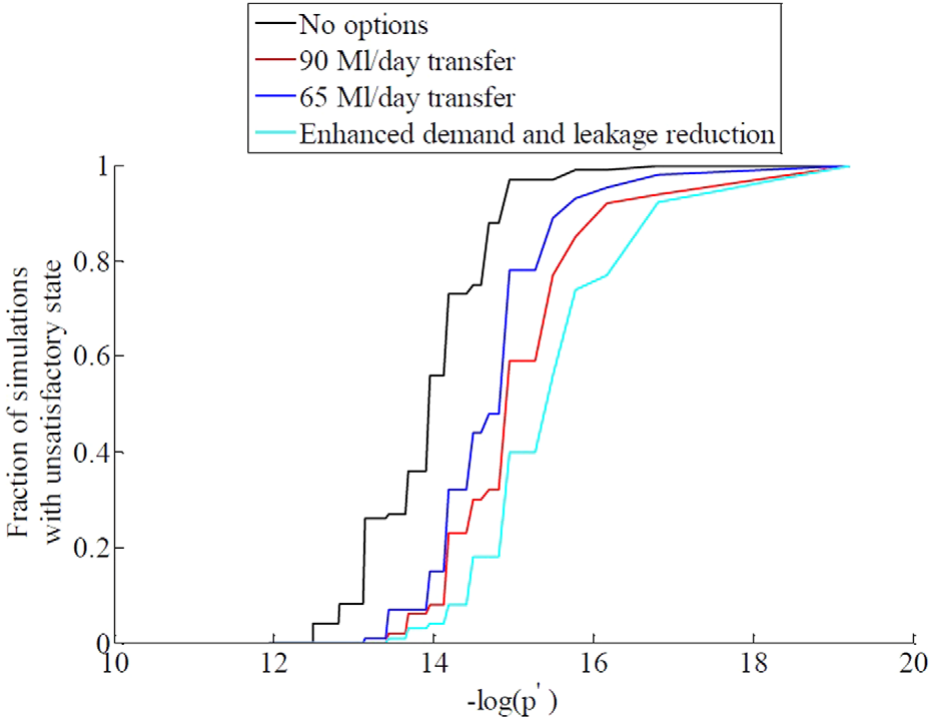
Worst system performance F_p'_curves for the water resource system in its current state (black line) and for three alternative drought management options.

This procedure was applied to each alternative option, and the resulting functions of *F_p′_* depicting system vulnerability were plotted against the log of the different *p*′ values in Figure 10. The vulnerability of the water system under a business as usual scenario is also plotted in Figure 10. The figure provides a means of visually comparing the options' vulnerability to droughts of gradually increasing duration and deficit. Low values of *F_p′_* plotted in Figure 10 extend far outside the drought events included in the Future Flows projections and, given the uncertainties in these projections, we are not suggesting that $p^{\prime }$ should be interpreted in probabilistic terms. The plot provides a concise means of comparing options' robustness (shown in Figure 8) in increasingly severe drought conditions.

Figure 10 shows that the enhanced leakage and earlier demand restriction option is less vulnerable for all values of $p^{\prime }$. The water transfers help to reduce vulnerability to drought compared to the no options scenario; however, their applicability in the case study area may be limited because droughts in the south of England show a high degree of spatial coherence [*Rahiz and New*, 2012]. Large regions encompassing more than one basin may experience the same drought, making water transfers between neighboring basins an unreliable drought management option unless long range transfers with very high costs and environmental impacts were to be considered [*Environment Agency*, 2006].

It should be noted that the ability of these alternative management options to reduce vulnerability is also a function of the system's demands. Future work will assess how vulnerability to drought changes given different assumptions about per capita consumption and population changes [e.g., *Singh et al*., 2015].

## 5 Discussion

Drought vulnerability analyses have traditionally been centered on prediction‐based approaches, which rely on the historical record and on estimates of future hydroclimatic conditions from climate models to forecast future drought occurrence and inform management actions. In this paper, we have shown that the use of a vulnerability‐based approach can provide greater insight into water resource system's response to drought and that climate model information is more useful and interpretable if presented within a vulnerability assessment framework where the effects of a wide range of hydroclimatic conditions on water resource system performance can be quantified. The coupling of a bottom‐up vulnerability analysis with climate projections proved to be a powerful tool to discover system's vulnerabilities and appraise the robustness of different adaptation options [e.g., *Brown et al*., 2012; *Steinschneider and Brown*, 2013; *Whateley et al*., 2014].

The approach presented in this paper is predicated on the hypothesis that future climate may bring about an increase in the risk of multiyear and more intense (i.e., higher deficit) droughts (e.g., the drought in California that began in 2012 [*Diffenbaugh et al*., 2015]) and that water managers need to test their system's vulnerability to these conditions and identify coping strategies. No attention was paid to identifying the physical processes that could cause the drought duration‐deficit conditions to which the water resource system is vulnerable. Droughts can be related to numerous physical processes, with short‐term droughts being associated to atmospheric circulation patterns such as anomalous stationary Rossby wave patterns [*Schubert et al*., 2011] and persistent anticyclones [*Peterson et al*., 2013], and longer and more extreme droughts being linked to longer‐term anomalies in sea surface temperature, for instance, those associated with ENSO [e.g., *Cook et al*., 2009; *Seager*, 2007]. The streamflow data considered in this study do not show any structured patterns in interannual variability or sensitivity to long‐term climate anomalies; however, applications of our approach in other parts of the world with strong interannual variability patterns may require extension of our method to account for periods of stronger or weaker monthly hydrological persistence.

More work is needed to understand which physical processes generate the drought conditions depicted in Figure 7, assess how the frequency and occurrence of these processes are going to change in a warming climate, and also analyze the relationship between hydrological drought duration and deficit and climate characteristics [e.g., *Van Loon et al*., 2014]. This latter point emphasizes the need to go beyond simply propagating climate model output into climate change vulnerability assessments toward a more refined understanding of the climatic and meteorological processes that lead to droughts and case‐by‐case application of climate model outputs of interest to decision‐makers [e.g., *James et al*., 2015].

In the case study application, we defined drought using a seasonal Q75 threshold. In temperate regions such as the UK, the variable threshold level method is the most widely used method to define hydrological drought [*van Huijgevoort et al*., 2012] and has been applied before in drought vulnerability studies [*Watts et al*., 2012]. Other hydrological studies employ similar seasonally varying flow quantiles as drought thresholds [*Tallaksen et al*., 2009; *Watts et al*., 2012]. Future work will investigate the extent to which our results change depending on the selection of the drought threshold.

The results are conditional on the bootstrapping strategy employed to resample monthly streamflow values. We employed this strategy to ensure the plausibility of the synthetic sequences and to avoid having to make assumptions on the streamflow distribution. As Figure 7 shows, bootstrapping the historical sequence has the disadvantage of not generating low flows beyond the historical range. More experimentation is required to explore whether or not hydrological drought scenarios can be generated using parametric distributions to sample the monthly streamflow values. While we are attracted by this possibility, we recognize that extreme low flows are often determined by in‐drought river regulation and reservoir operation, as well as complex groundwater interactions, so may not be amenable to simple statistical treatment.

In the water resource system simulation, the output from groundwater sources was assumed to be constant. Future work will test the system's response during a drought under different levels of groundwater output in order to understand the potential role of groundwater depletion in leading the system into an unsatisfactory state. The change in stakeholders' expectations with respect to moderating drought impact on aquatic ecosystems—which could lead to a decrease in public water supply abstraction rights—is another important aspect that was not considered in this study [cf. *Marsh*, 2007].

## 6 Conclusions

The reality of climate change and the projected intensification of the hydrological cycle mean that water resources managers need to understand their systems' ability to cope with a wide range of drought conditions. However, obtaining information about future drought characteristics is difficult given the limited historical record, the uncertainty surrounding hydroclimatic projections at a regional scale and the weakness of climate models in representing precipitation persistence. In this study, we presented a vulnerability‐based approach using copulas to generate synthetic streamflow sequences and to provide water managers with a technique to evaluate water management options vulnerability to a wide range of hydrological drought duration and deficit conditions and different levels of monthly hydrological persistence.

The method is based on the use of copulas to characterize and perturb the autocorrelation structure of monthly streamflow sequences, which determines drought duration and deficit. The copula approach provides a flexible means of exploring a continuous range of possible drought conditions, including droughts with much greater severity than the observed record that are still consistent with the monthly streamflow hydrology.

The synthetic streamflow series were used to test London's water supply system vulnerability to drought duration and deficit. Results from the case study indicate that London's water supply system is vulnerable to intense droughts with deficits greater than 1.5 × 10^4^ m^3^ and durations greater than 18 months. Although the system was shown to be able to cope with historical drought variability, we find that a drought of similar length to the worst drought on record but with greater deficit could increase the chances of the system reaching an unsatisfactory state (i.e., severe water use restrictions). Indeed we find that the system is more sensitive to severe deficit than it is to, proportionately equally severe, increases in drought duration beyond the worst conditions in the observed record. We demonstrate that the water resource system is sensitive to monthly hydrological persistence characteristics, suggesting that changes in this critical variable should be considered in climate change water resource vulnerability assessments.

The vulnerability‐based approach presented here can benefit water management decision‐making in several ways. It provides insight into the drought conditions that lead the water resource system to unsatisfactory performance and into the vulnerability of management options to drought conditions. Most climate change impact assessments in water resources—with the exception of *Steinschneider et al*. [2015]—normally represent climate change using additive or scalar perturbations to observed statistics (delta change or change factor methods), which downplay possible future changes in interannual hydrological variability and neglect changes to streamflow statistics that are critical to water resource system reliability [*Johnson and Sharma*, 2011]. Our method allows water managers to include the effects of changing levels of monthly hydrological persistence in their climate change impact assessments.

Another benefit of our approach is that it provides a drought vulnerability space onto which current hydroclimatic projections can be mapped. This facilitates the communication of climate model outputs to water managers by projecting the impact on decision‐relevant variables such as the likelihood of reaching an unsatisfactory state and provides a framework that can be easily updated as new hydroclimatic projections become available. Furthermore, in our framework, climate model information can be coupled with the vulnerability analysis to compare alternative water management options in terms of their vulnerability to increasingly severe and long drought conditions. We have done this by developing a vulnerability metric to explore system sensitivity to droughts of increasing deficit and duration.

The method was applied to an urban water resource system, but it could be equally applied to assess the vulnerability of aquatic ecosystems, hydropower production, or irrigation systems to different hydrological drought conditions, as long as suitable system thresholds can be identified. Furthermore, the approach can be upscaled spatially to account for and model the spatial coherence of drought phenomena, and it is envisioned that this task will be performed in the future.

## Supporting information

Suppporting Information S1
