# Supplementary material for: Assessing water resource system vulnerability to unprecedented hydrological drought using copulas to characterize drought duration and deficit
Source: Water Resour Res. 2015 Nov 16;51(11):8927–48. doi: 10.1002/2015WR017324 (PMC4991281; doi:10.1002/2015WR017324)
Supplement: Supplementary file 1 — Suppporting Information S1 [file WRCR-51-8927-s001.doc]

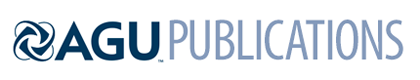


*Water Resources Research*

Supporting Information for

**Assessing water resource system vulnerability to unprecedented hydrological drought using copulas to characterize drought duration and deficit**

Edoardo Borgomeo, Georg Pflug, Jim W. Hall, Stefan Hochrainer-Stigler

University of Oxford

**Contents of this file**

Text S1 to S

Figures S1 to S14

**Introduction**

This supporting information presents scatter plots of the monthly streamflow observations for consecutive months for the Thames at Kingston (1883-2012), plots of K_n_ and a plot of the Gumbel copula fitted to the Future Flows drought duration and deficit data.

S1. Scatter plots of monthly streamflow observations for consecutive months

To illustrate the nonlinear temporal dependence in the monthly streamflow observations for the Thames at Kingston, we shown 12 scatter plots, one for each pair of consecutive months.


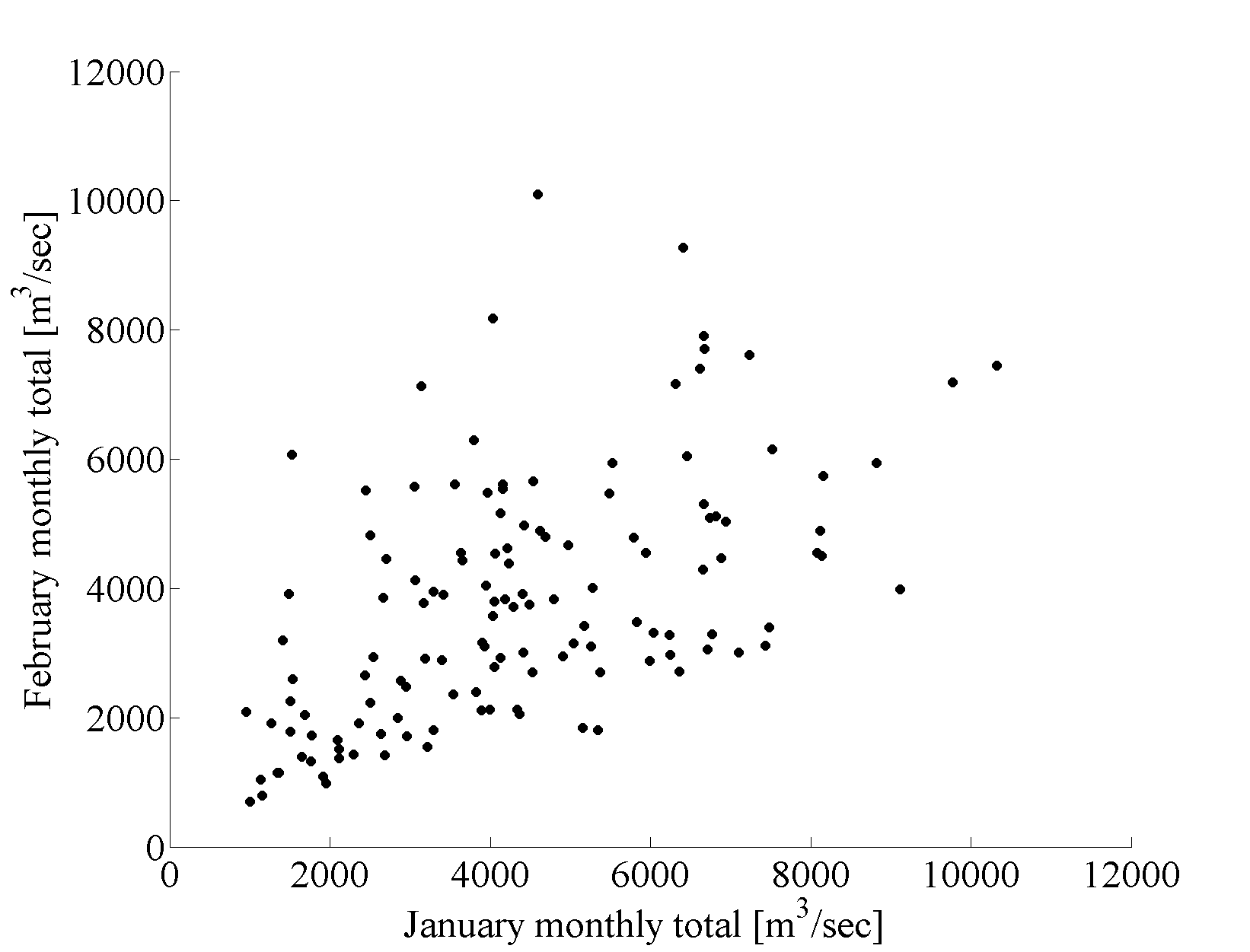


**Figure S1**. Scatter plot of the January and February monthly totals observed for the Thames at Kingston (1883-2012).


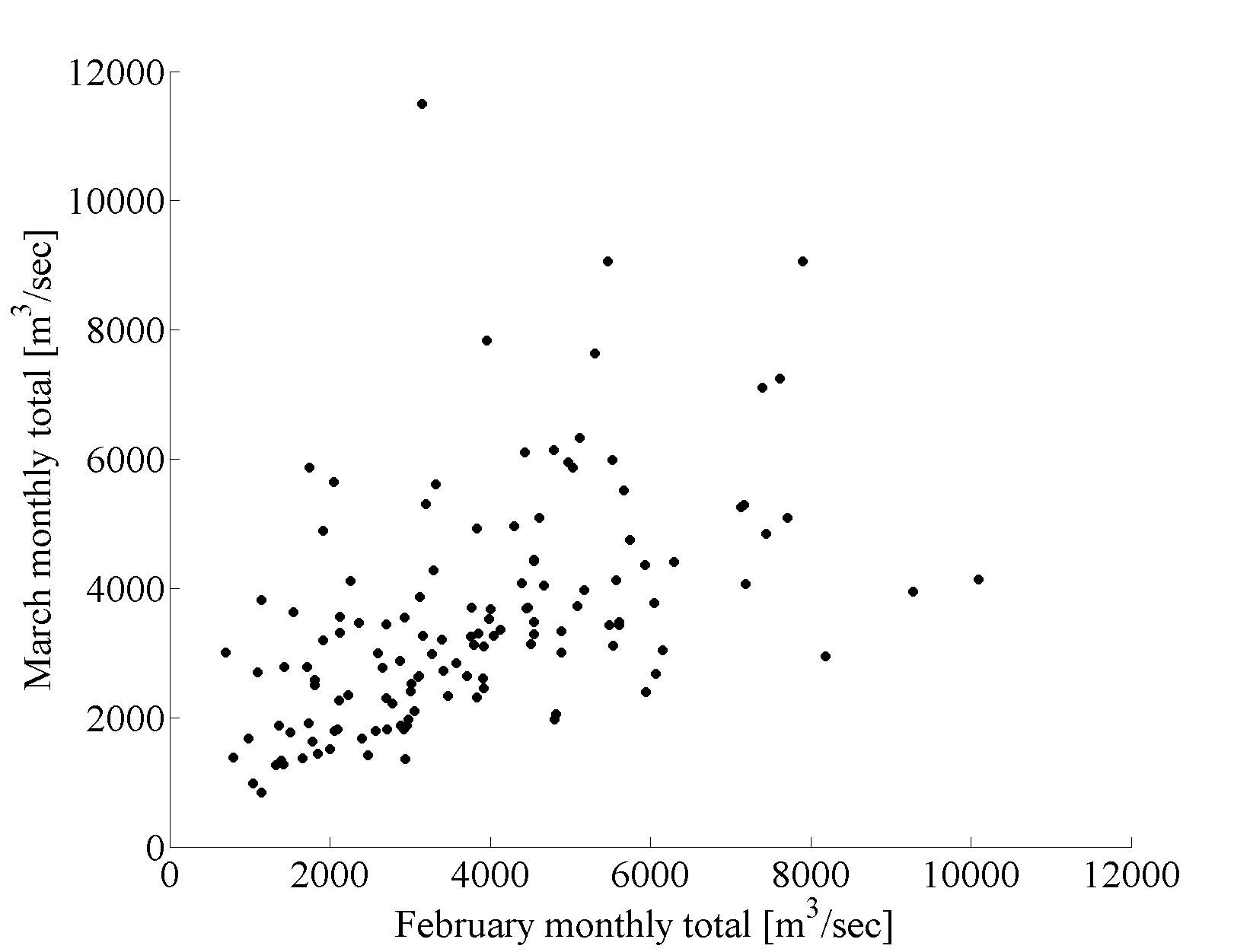


**Figure S2**. Scatter plot of the February and March monthly totals observed for the Thames at Kingston (1883-2012).


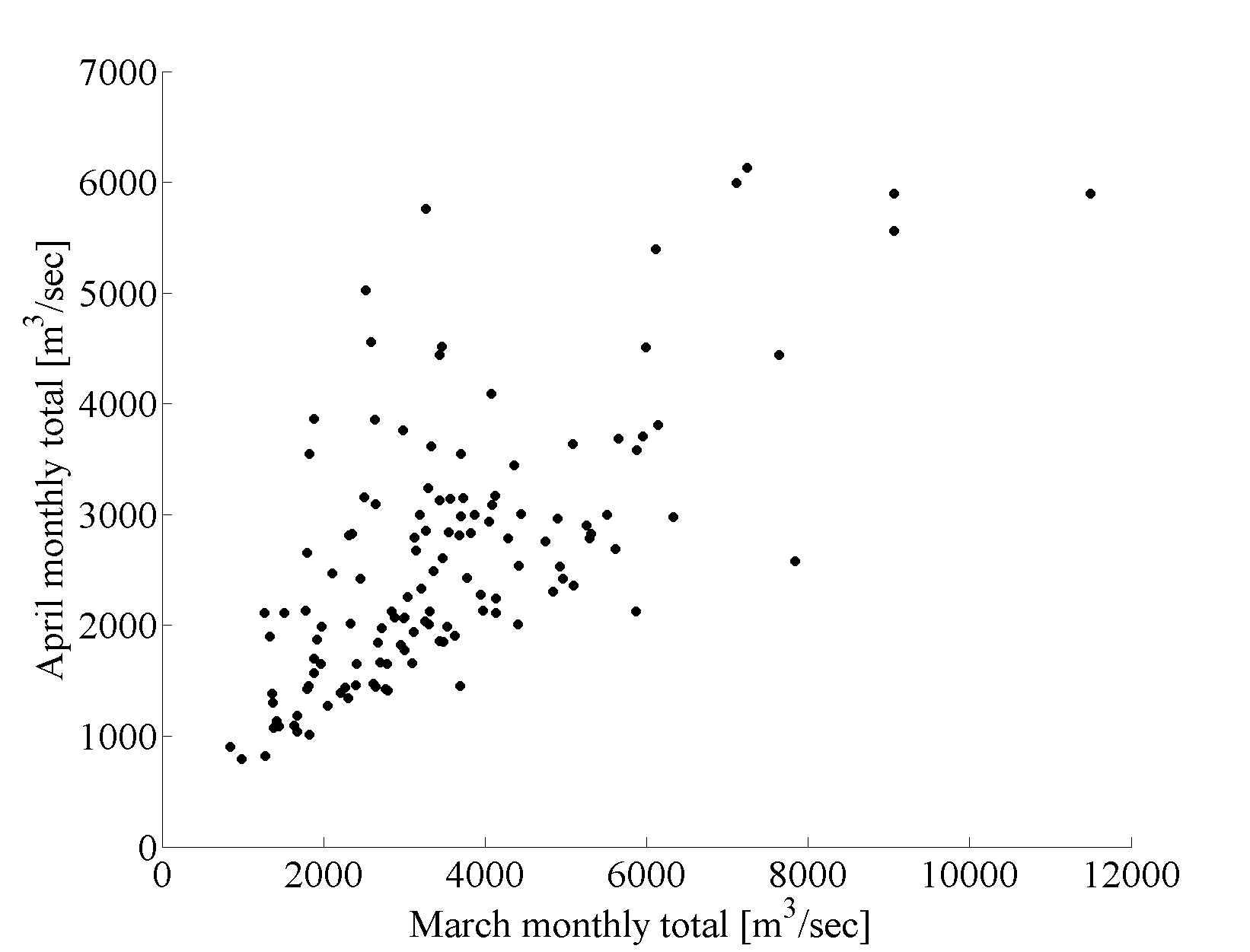


**Figure S3**. Scatter plot of the March and April monthly totals observed for the Thames at Kingston (1883-2012).


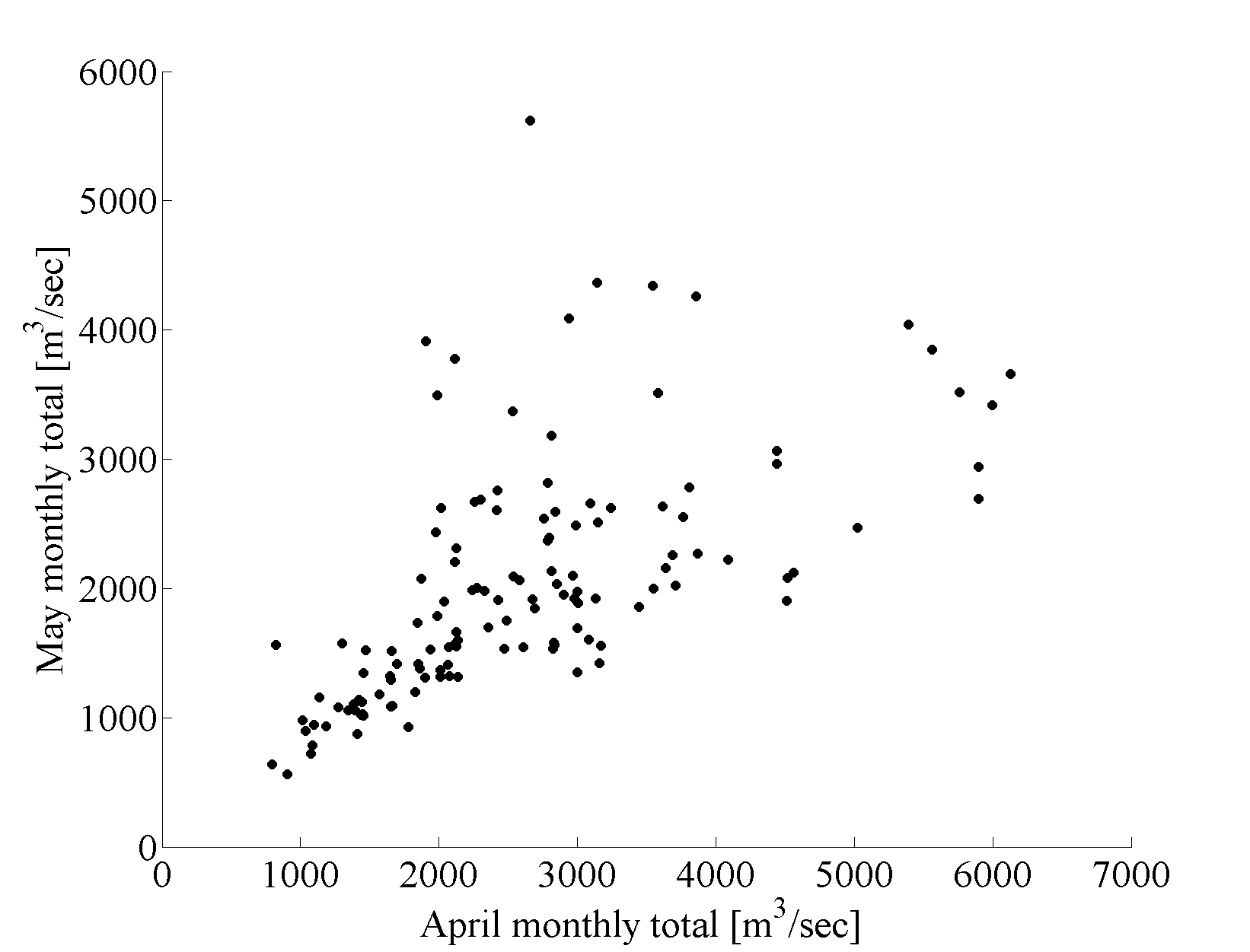


**Figure S4**. Scatter plot of the April and May monthly totals observed for the Thames at Kingston (1883-2012).


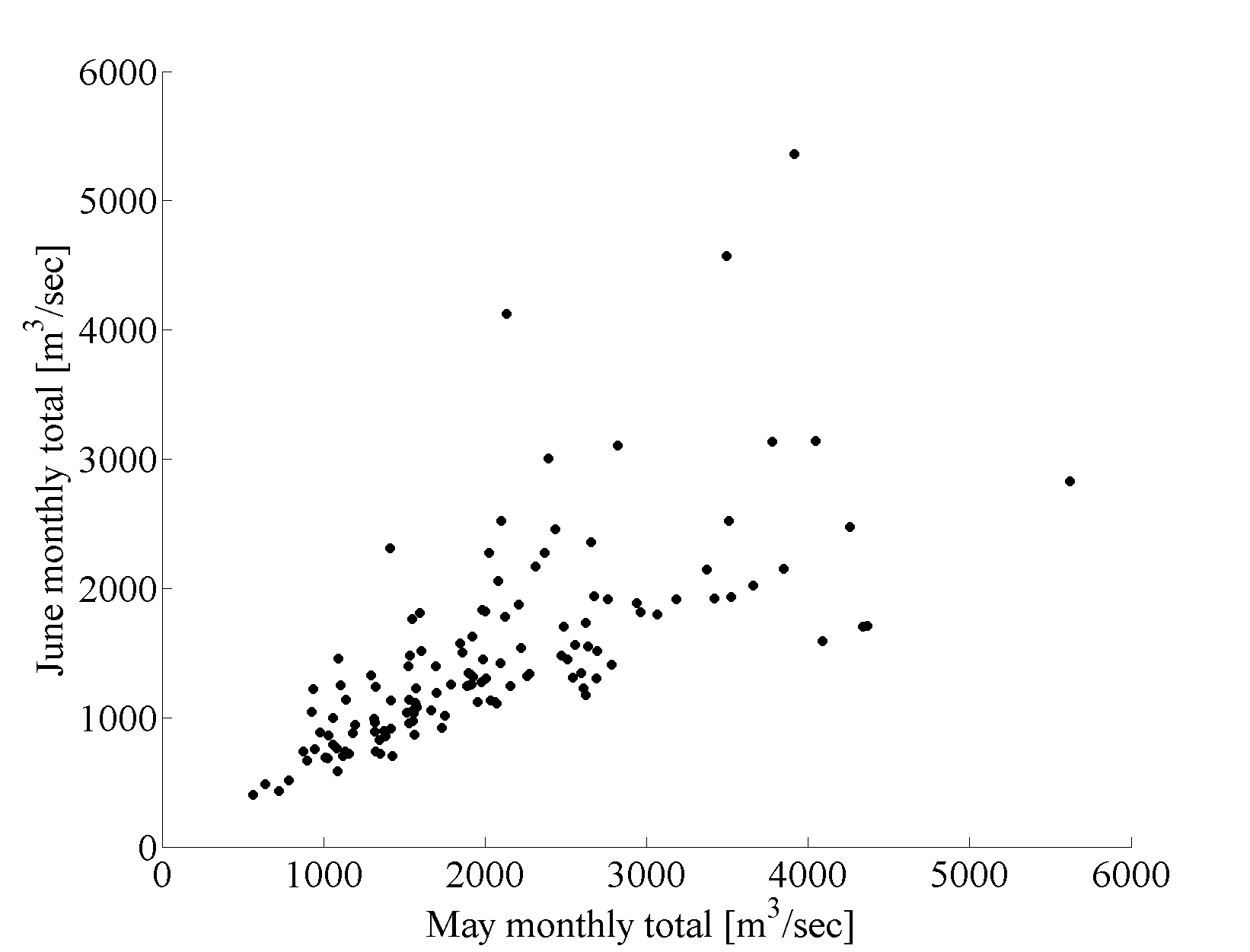


**Figure S5**. Scatter plot of the May and June monthly totals observed for the Thames at Kingston (1883-2012).


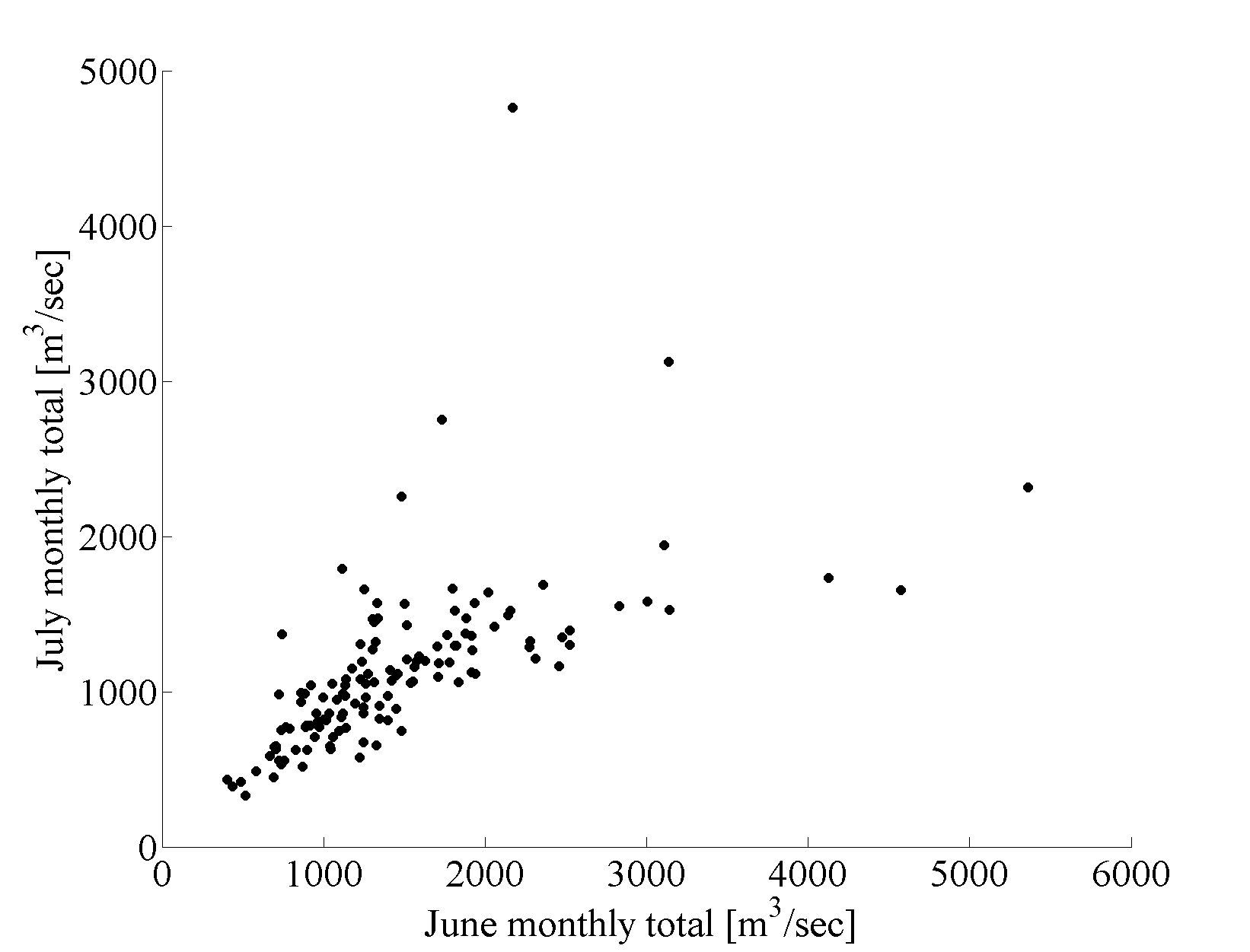


**Figure S6**. Scatter plot of the June and July monthly totals observed for the Thames at Kingston (1883-2012).


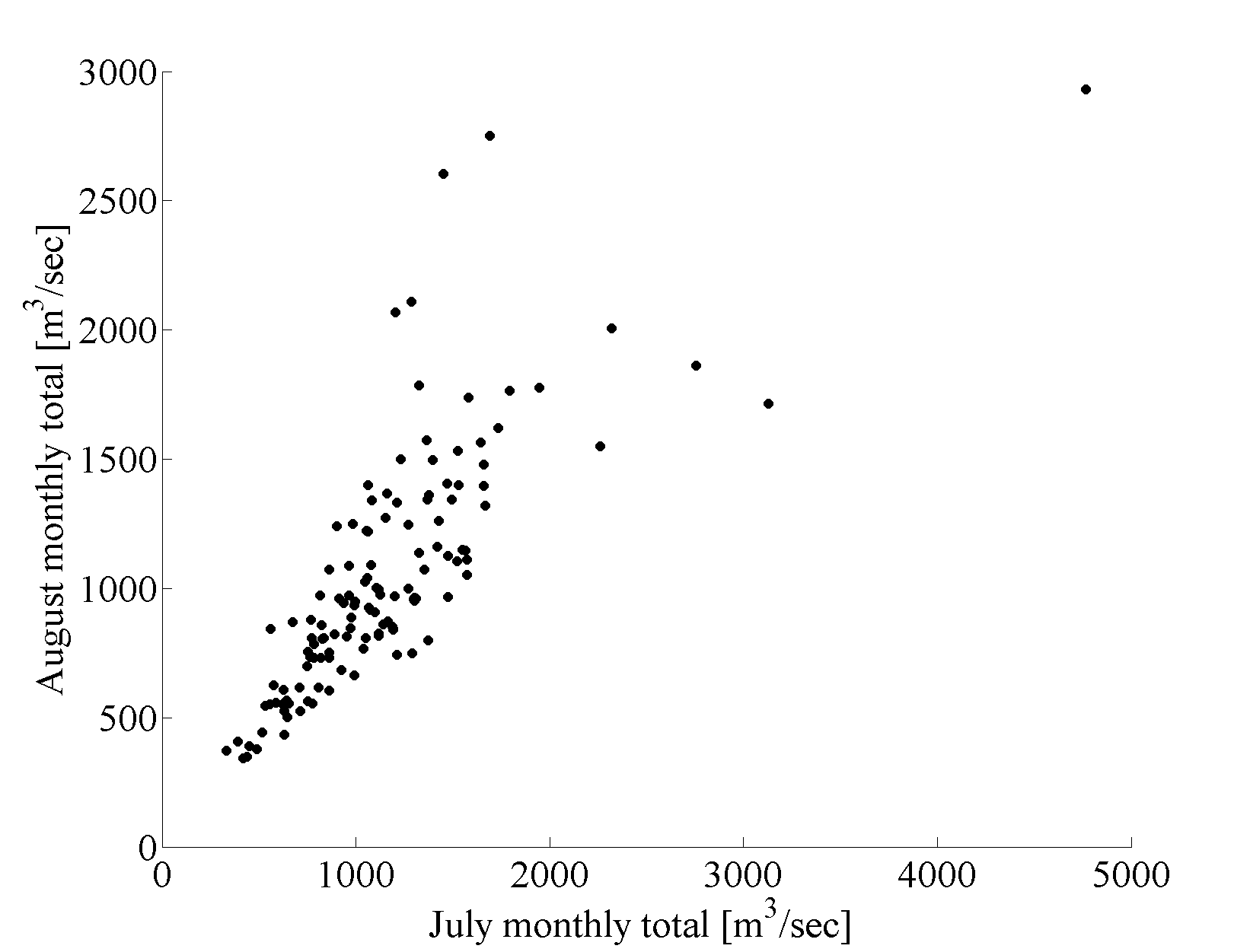


**Figure S7**. Scatter plot of the July and August monthly totals observed for the Thames at Kingston (1883-2012).


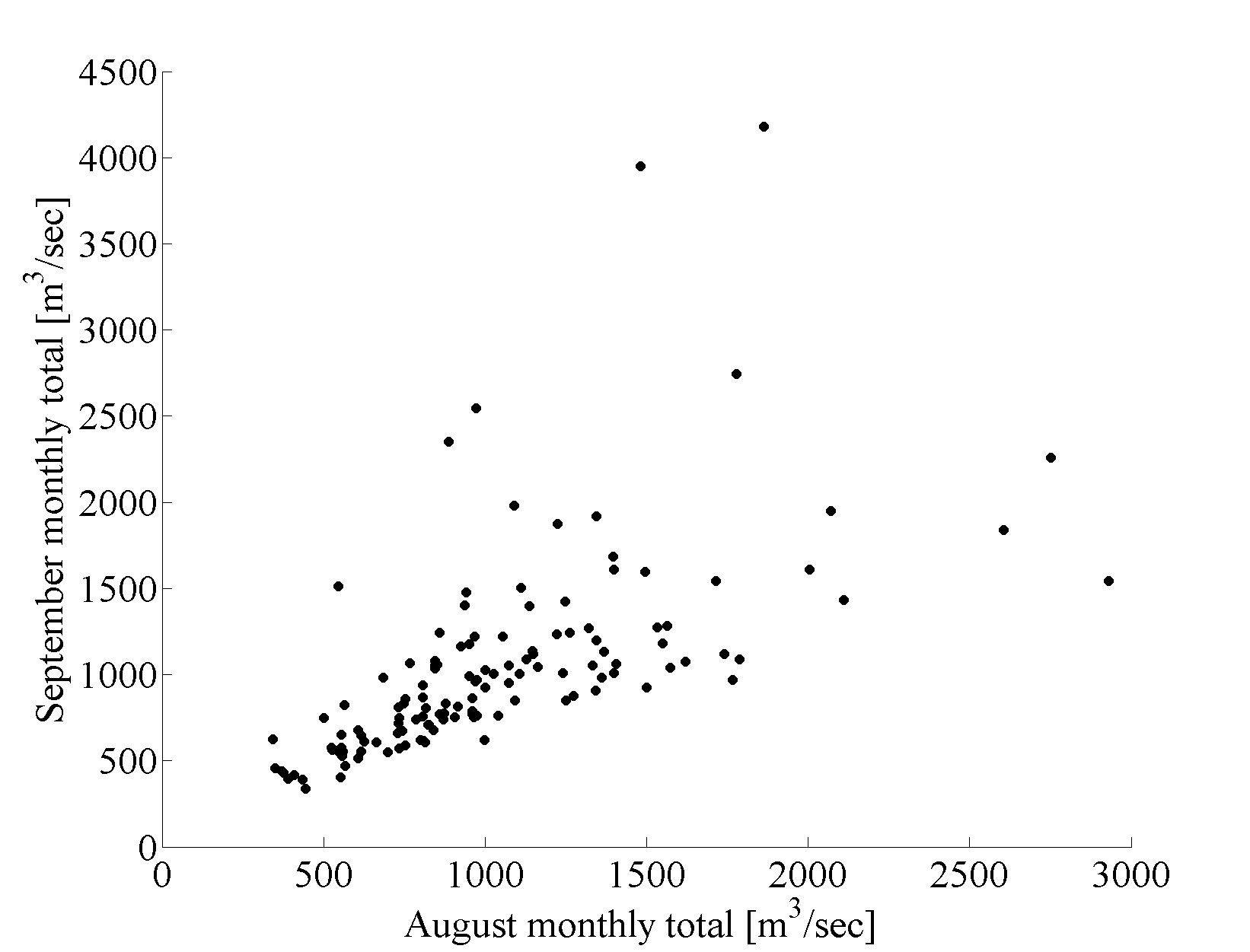


**Figure S8**. Scatter plot of the August and September monthly totals observed for the Thames at Kingston (1883-2012).


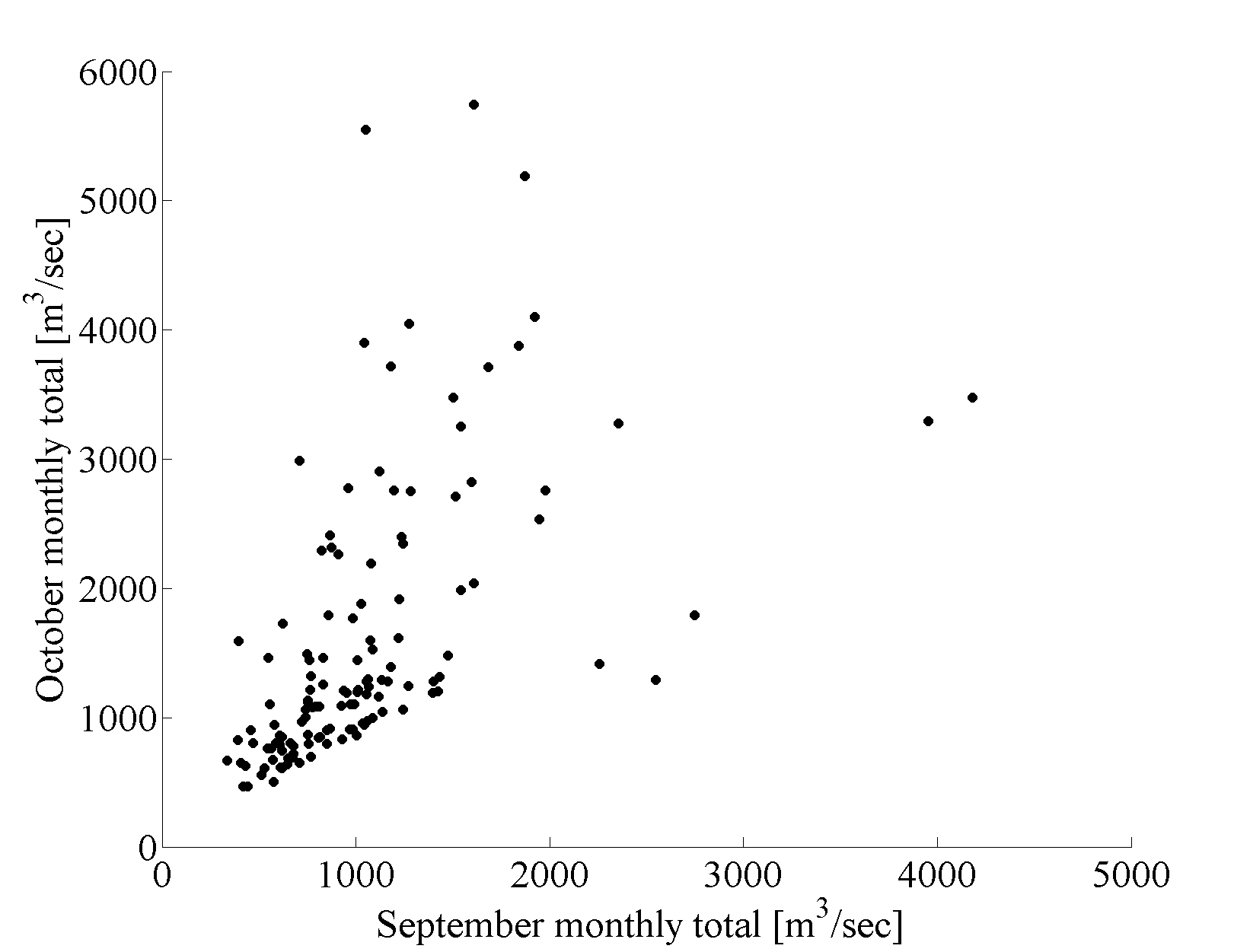


**Figure S9**. Scatter plot of the September and October monthly totals observed for the Thames at Kingston (1883-2012).


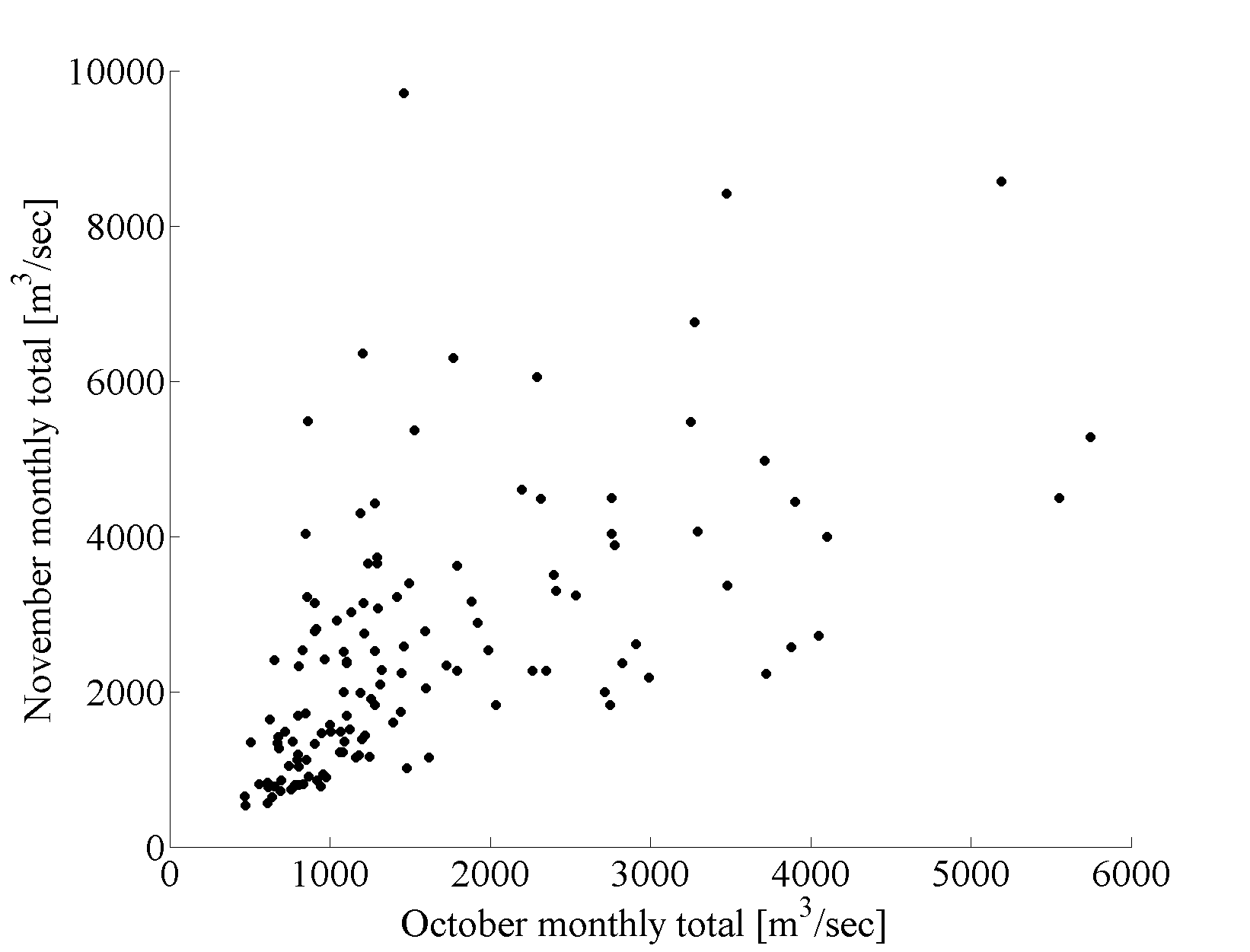


**Figure S10**. Scatter plot of the October and November monthly totals observed for the Thames at Kingston (1883-2012).


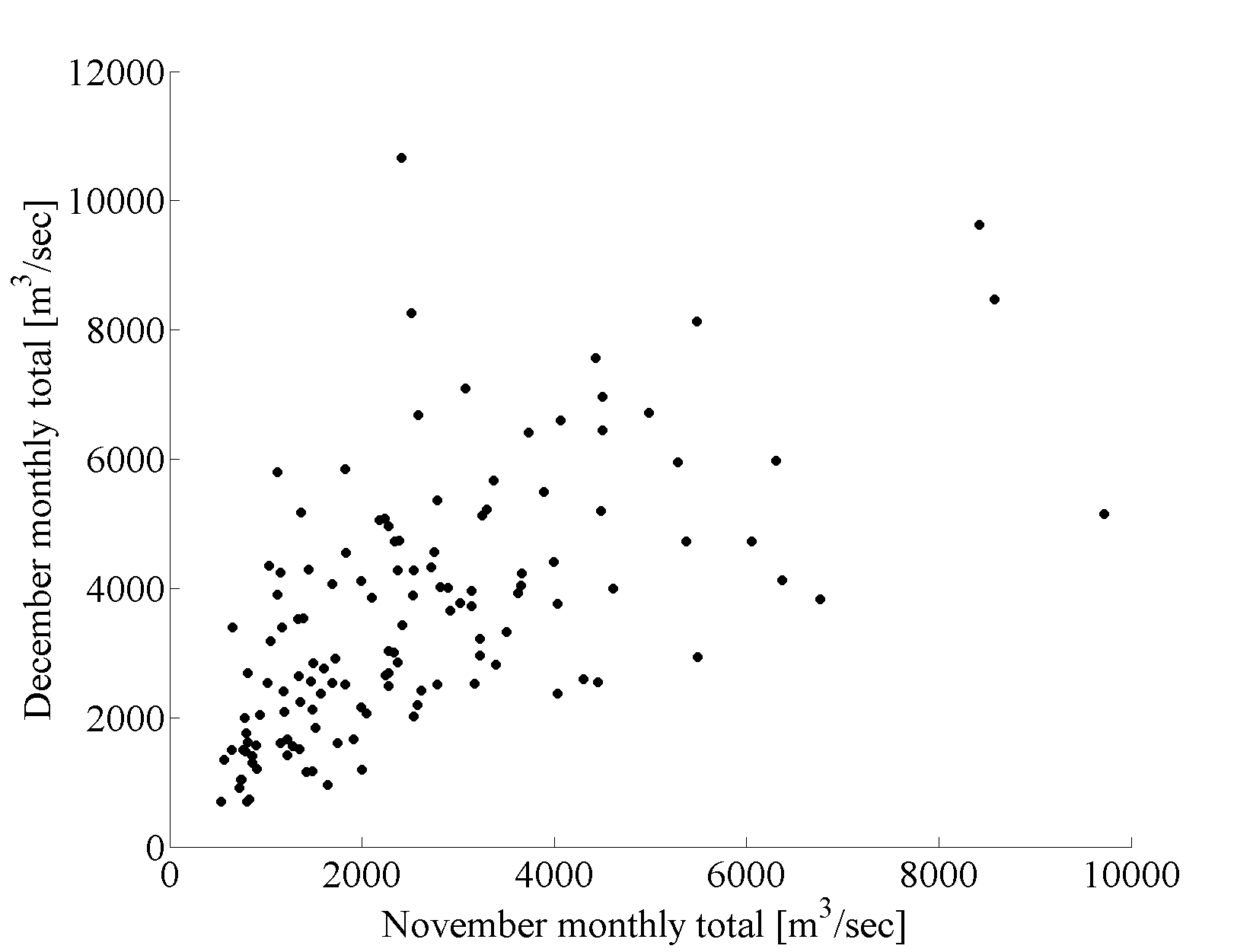


**Figure S11**. Scatter plot of the November and December monthly totals observed for the Thames at Kingston (1883-2012).


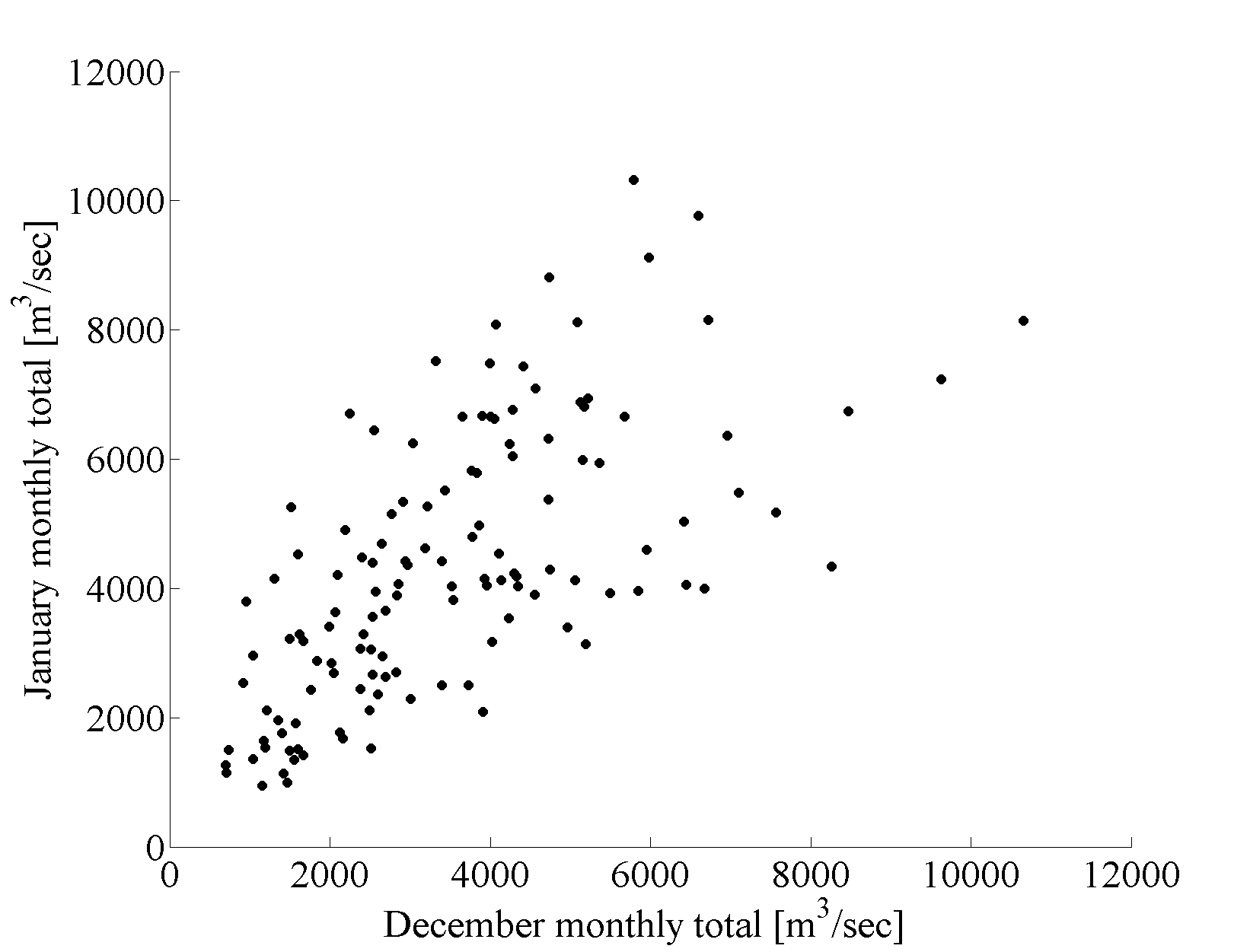


**Figure S12**. Scatter plot of the December and January monthly totals observed for the Thames at Kingston (1883-2012).

S2. K_n_ plots

The empirical copulas C(Y_i-1_,Y_i_) obtained for each month pair were compared in Figure S13 with the fitted theoretical Clayton and Frank parametric copulas C_θ_(Y_i-1_,Y_i_) with the estimated parameters shown in Table 3. The circles in Figure S13 indicate the empirical distribution K_n_ as defined in equation 7, and the solid red and dotted black lines show the fitted Clayton and Frank copulas, respectively. The concave shape in the plots in Figure S13 indicates a positive relationship between monthly streamflow totals from consecutive months. A good fit is obtained for both Clayton and Frank copulas and visual judgment alone cannot be used to select the appropriate copula.

Figure S13. Graphs of K_n_ and K_θ_ for the consecutive total monthly flows for the River Thames at Kingston. Points indicate empirical values K_n_, red curve indicates the Clayton copula values and the dotted line indicates the Frank copula values.

S3. Fitting a Gumbel copula to the drought duration and deficit projections

The marginal distributions of the drought duration and deficit of the Future Flows were found to be exponentially distributed and the data transformed with the inverse exponential distribution are shown in Figure S14. The Gumbel copula was found to best represent the joint distribution of duration and deficit, and contours for this copula are also shown in Figure 9.
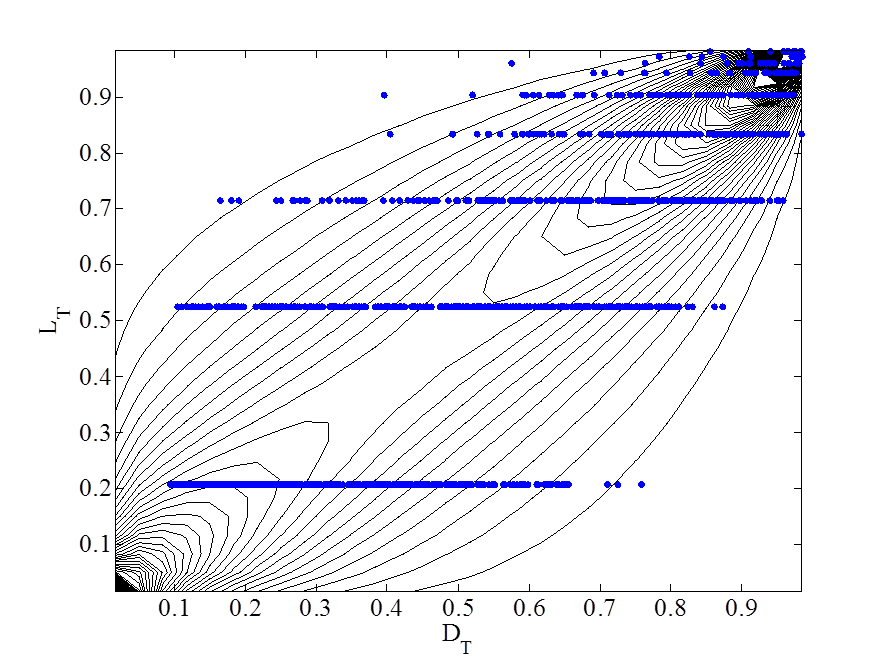


Figure S14. . Scatter plot of the transformed drought duration L_T_ and deficit D_T_ data (blue dots) and density plot of Gumbel’s copula with parameter θ = 2.7.
